# MolecureAI: an agentic AI platform for autonomous drug discovery

**DOI:** 10.3389/fphar.2026.1917774

**Published:** 2026-09-29

**Authors:** Mohamed Amine Chadi, Asmaa Amzil, Hajar Ezzahoud, Hajar Mousannif

**Affiliations:** Laboratory of Computer Science and Smart Systems (LISI), Faculty of Sciences Semlalia, Cadi Ayyad University, Marrakech, Morocco

**Keywords:** ADMET safety gating, autonomous drug discovery, benchmark target validation, campaign memory, cost-ordered cascade, LLM agent, molecular generation, tool orchestration

## Abstract

Accelerating the identification of viable drug candidates across therapeutic areas demands autonomous systems for molecular generation that enforce chemical correctness by structural invariants rather than model capability. We present MolecureAI, an autonomous AI agent architecture for closed-loop drug discovery with cost-ordered cascade evaluation that addresses four capability gaps unresolved across the field: autonomous design strategy selection, persistent cross-campaign structural memory, in-loop ADMET safety gating, and literature-scale evidence integration into the generative loop. A cost-ordered cascade concentrates full structure-based evaluation on a small pre-filtered survivor set, achieving a 2.5× reduction in candidates advanced relative to those generated. An in-loop metabolic gate enforces Absorption, Distribution, Metabolism, Excretion, and Toxicity (ADMET) safety by writing flagged reactive substructures to a per-target learned state, suppressing identified liabilities from all subsequent generation rounds. A persistent campaign memory influences generation trajectories by embedding prior structural success and failure patterns directly into the design context. We validate these architectural invariants across three structurally diverse benchmark targets (EGFR, ${\text{KRAS}}^{\text{G}12\text{C}}$ , and BRD4) through a five-condition component ablation and a controlled memory ablation, and confirm generator agnosticism across four language-model backends *via* a deterministic recalibration layer that overrides backend self-evaluation bias. Without explicit chemotype instruction, the pipeline computationally converged toward candidates bearing the 4-anilinoquinazoline scaffold characteristic of the approved inhibitors erlotinib and gefitinib, from structural feedback alone. Persistent campaign memory showed directional but non-significant trends toward lower inter-round score variance and higher late-stage advance rate ( n≈90 per condition; all p>0.20 ). The component ablation reveals that optimizing for a single objective collapses performance on others; only full orchestration resolves this tension. Advancement rates above 79% across all backends confirm that the validated properties are generator-agnostic, enforced by architectural invariants rather than model capability. All reported outcomes are properties of the computational generation process; prospective biochemical validation of the advanced candidates remains the primary direction for future work.

## 1 Introduction

Bringing a single small-molecule therapeutic from target identification to clinical approval requires, on average, more than a decade of iterative research and an investment exceeding two billion dollars, with a clinical trial failure rate surpassing ninety percent (Scannell et al., 2012; Roland et al., 2024; DiMasi et al., 2016). Approximately forty percent of late-stage clinical failures are attributable to insufficient efficacy and a further thirty percent to safety and toxicity liabilities, problems that are, in principle, detectable at the molecular level well before a compound enters the clinic (Scannell et al., 2012; DiMasi et al., 2016). Closing this gap computationally requires systems that not only generate candidate molecules but reason about strategy, safety, and evidence the way an expert medicinal chemist does across an entire hit-to-lead campaign, not just within a single generative step.

Computational molecular generation has progressively advanced toward structure-aware, multi-objective methods along four largely separate lines. Causal language models such as MolGPT (Bagal et al., 2022) and DrugGPT (Li et al., 2023) demonstrated high chemical validity in generated candidates. Three-dimensional equivariant diffusion models, including TargetDiff (Guan et al., 2023) and DiffSBDD (Schneuing et al., 2024), condition generation directly on binding-pocket geometry. Reinforcement learning platforms such as REINVENT 4 (Loeffler et al., 2024) and DrugEx v2 (Liu et al., 2021), together with conditional loss-reduction approaches to *de novo* generation (Chadi et al., 2022), optimize simultaneously for potency and ADMET objectives. A parallel line of work has explored text- and target-conditioned generation, where molecular structures are guided by natural-language descriptions or chemical concept embeddings rather than by explicit property objectives; representative examples include LDMol (?), TGM-DLM (Gong et al., 2024), and VeGA-RX/VeGA-SCX (Delre et al., 2026). MolecureAI’s novelty lies not in this conditioning paradigm but in the agentic orchestration layer and closed-loop evaluation workflow that surrounds generation. Fragment-based drug discovery and compound repurposing have similarly received dedicated computational attention (Erlanson et al., 2016; Murray and Rees, 2012; Pushpakom et al., 2019), and metabolic liability assessment has been addressed by tools such as BioTransformer (Djoumbou-Feunang et al., 2019), MetaSite (Cruciani et al., 2005), and FAME 3 (Šícho et al., 2019). Separately, LLM-based tool-augmented agents have emerged as a fourth line: ChemCrow (Bran et al., 2024) showed that equipping an LLM with expert-designed chemistry tools enables autonomous accomplishment of tasks spanning organic synthesis, drug discovery, and materials design, and the Model Context Protocol (MCP) (Anthropic, 2024) subsequently generalized this into a standardized, generator-agnostic interface between LLMs and external computational tools. A growing body of MCP- and agent-based drug discovery platforms has followed, spanning early closed-loop cheminformatics toolsets (McNaughton et al., 2024; Ansari et al., 2024), autonomous navigation across clinically relevant targets (Averly et al., 2025), trajectory-aware lead refinement (Li et al., 2026), the first serious case for cross-campaign memory as a first-class architectural element (Wang et al., 2026), end-to-end systems integrating database, tool, and model layers (Pan et al., 2025; Cao et al., 2026; Yousefi et al., 2025), industrial deployment (He et al., 2025), and domain-agnostic agents generalizing across biological reasoning tasks (Huang et al., 2025; Gao and Zitnik, 2025). Table 1 compares MolecureAI with representative systems from two complementary perspectives. Panel A positions MolecureAI architecturally against five systems, ChemCrow (Bran et al., 2024), REINVENT 4 (Loeffler et al., 2024), DrugEx v2 (Liu et al., 2021), TargetDiff (Guan et al., 2023), and MolMem (Wang et al., 2026), along four axes answerable from published system design: generation and strategy, evaluation and safety, memory, and tool architecture. Panel B adds a focused comparison against ChemCrow alone, the only one of the five that shares MolecureAI’s generative paradigm as an LLM-driven tool-use agent rather than a purpose-built generative or reinforcement-learning model; the remaining four differ too fundamentally in output type, training requirement, and evaluation protocol for a quantitative comparison to isolate architectural contribution, and are therefore compared at the architectural level only. Panel B provides contextual evidence against the closest available LLM-agent baseline under each system’s default operating configuration (Section 2.10), rather than a controlled, backend-matched benchmark, which is identified as a priority for the next evaluation cycle (Section 4.7).

**TABLE 1:** Comparison of molecureai with existing drug discovery systems. (A) Architectural comparison against five representative systems. (B) Focused comparison against ChemCrow on EGFR, provided as contextual evidence against the closest available LLM-agent baseline rather than a controlled benchmark (Section 2.10).

(A) Architectural comparison				
System	Generation and strategy	Evaluation and safety	Memory	Tool architecture
MolecureAI	LLM-driven SMILES generation with autonomous strategy selection (FBDD, lead optimization, repurposing)	Cost-ordered cascade (property → ADMET/metabolic → docking → pose); target-specific ChEMBL-derived thresholds	Three persistent stores: evaluation history, per-target learned state, regulatory priors	MCP tool layer; generator-agnostic across 4 LLM backends
ChemCrow (Bran et al., 2024)	LLM agent for synthesis planning, not molecule generation	Tool-based workflow; no staged validation cascade	Not supported	18 expert-designed chemistry tools
REINVENT4 (Loeffler et al., 2024)	RNN/transformer generation *via* RL and curriculum learning	User-defined scoring function only; no in-loop metabolic or cardiac gate	Not supported (configuration is run-scoped)	Not supported (standalone generative framework)
DrugExv2 (Liu et al., 2021)	RL framework for *de novo* design	Multi-objective scoring and reward optimization; no staged validation cascade	Not supported	Not supported (standalone RL generator)
TargetDiff (Guan et al., 2023)	SE(3)-equivariant diffusion conditioned on pocket geometry	Vina-based affinity only; no in-loop safety screening	Not supported	Not supported (self-contained model)
MolMem (Wang et al., 2026)	RL-based molecular optimization	Reward-driven optimization with memory feedback; no validation cascade or safety screening	Cross-campaign optimization memory (no regulatory layer)	Not supported (optimization-focused framework)

(B) Focused comparison vs. ChemCrow: EGFR, default operating configuration		
Metric	MolecureAI	ChemCrow
LLM backend	Llama 3.1 8B Instant (Groq)	GPT-4o (OpenAI)
Generation mode	Iterative (27 iterations → 30 advances)	Single-shot (30 requested)
Valid unique candidates	30/30	18/30
ADMET pass rate	100% (30/30)	0% (0/18)
Docking pass ( ≤−7.0 kcal/mol)	100% (30/30)	77.8% (14/18)
Mean docking score	−7.82 kcal/mol	−7.50 kcal/mol
Final hits (ADMET + docking)	30	0
Runtime/est. API cost	∼ 91 min/$0.12	∼ 18 min/$0.09

What every one of these lines shares, however, is a boundary drawn around a single campaign: the reward function is user-supplied, the scoring components are fixed before the run, and every molecule and every lesson is discarded when the run ends. Four specific gaps follow from this shared limitation. The gap is strategic: no published autonomous platform decides between fragment growth, lead optimization, and repurposing from the target’s own evidence; strategy remains a hyperparameter the user must set, which assumes the user already knows the answer the system was meant to provide. It is structural and temporal: outside MolMem, chemical knowledge does not persist across campaigns, and even MolMem has not demonstrated shared structural knowledge between independent researchers working on the same target; existing systems also apply universal drug-likeness filters rather than grounding property thresholds in the empirical bioactivity distributions of known actives for specific targets (Mendez et al., 2019), and structural validation has not been systematically extended beyond predicted binding affinity to include binding pose geometry, active-site constraints, pocket occupancy, or pLDDT/B-factor confidence scores. It is metabolic: safety tools such as BioTransformer (Djoumbou-Feunang et al., 2019) operate as *post hoc* filters disconnected from the generation loop, so a metabolic alert raised after a candidate list is fixed cannot stop the generator from proposing the same liability again. And it is epistemic: no existing platform integrates literature-scale evidence synthesis directly into the design loop, so regulatory context, bioactivity precedent, and intellectual property landscape must be assembled manually and remain disconnected from generative decisions. Systems that address one of these gaps tend to leave the others open, and none combines autonomous strategy selection, persistent structural memory, and in-loop metabolic gating within a single deployable platform.

MolecureAI is an autonomous, natural-language drug discovery platform addressing these four gaps in a unified architecture. The platform is publicly accessible at https://www.molecureai.com/as a deployed web application, where registered users can run molecular design campaigns through the full cascade pipeline. Target resolution is delegated to the OrigeneMCP tool ecosystem (Zhang et al., 2025); everything from compound retrieval through strategy selection, in-loop evaluation, routing, planning, and persistent memory is MolecureAI’s contribution. This paper specifies the scientific design of the system and the rationale for each architectural decision. Not every architectural claim made here carries the same evidentiary weight: some contributions are directly validated by the experiments reported in this work, others are implemented and mechanistically motivated but await independent benchmarking, and this distinction, rather than being left for the reader to infer, is stated explicitly for every contribution in Table 2 (Section 2.10) and carried through the Results and Discussion that follow.

**TABLE 2:** Evidentiary status of the architectural contributions and core claims. This table covers experimentally validated contributions and implemented but not yet benchmarked features; quantitative outcomes are reported in Section 3 and Section 4. Planned future capabilities not yet implemented are described in Section 4.8.

Contribution	Protocol basis	Status
Cost-ordered cascade and multi-tier structural validation	EGFR, ${\text{KRAS}}^{\text{G}12\text{C}}$ , BRD4; full pipeline and five-condition ablation. Tier-4 pose-level validation only; AlphaFold fallback was not exercised.	Experimentally validated (Partial)
Integrated metabolic and cardiac safety gating	BioTransformer profiling applied across experiments 1 and 2.	Experimentally validated
Persistent CampaignMemory	EGFR only; With/Without memory ablation (experiment 3).	Experimentally validated
Generator agnosticism	EGFR; 4 backends (groq-8b, claude-sonnet, gpt-4o, gemini-1.5-pro).	Experimentally validated
Autonomous design strategy selection	Applied consistently across all campaigns.	Architecturally implemented
Research campaign synthesis	Not exercised in the present empirical study.	Architecturally implemented

Five architectural contributions are introduced in this paper. (1) Autonomous design strategy selection, a system-level decision computed from five orthogonal evidence signals before the first generative step, replacing the strategy-as-hyperparameter assumption (Section 2.2). (2) Target-specific property thresholds derived from empirical bioactivity distributions rather than universal drug-likeness heuristics (Mendez et al., 2019), combined with multi-tier structural validation extending assessment beyond docking scores to include binding geometry, active-site constraints, and pocket occupancy (Section 2.4). (3) Integrated metabolic and cardiac safety gating through BioTransformer (Djoumbou-Feunang et al., 2019), positioned as a core design feature rather than a post-processing filter, closing the feedback loop that prior systems leave open (Section 2.5). (4) A unified generation and research tool layer in which literature-scale evidence synthesis constrains design directly (Sections 2.8 and 2.7). (5) A shared structural memory architecture accumulating typed, structurally indexed lessons across independent discovery sessions (Section 2.6). Table 2 states precisely which of these five are directly validated, which are validated only in part, and which are implemented but not yet independently benchmarked in the present study, so that this boundary is transparent from the outset rather than discovered piecemeal in later sections.

## 2 Materials and methods

MolecureAI is organized as a pipeline of five functional layers, each with a distinct responsibility, and the architecture is designed so that no layer can be bypassed or short-circuited by any other. Figure 1 maps the complete end-to-end flow, and the five layers it depicts are the organizing principle of this section.

**FIGURE 1:**
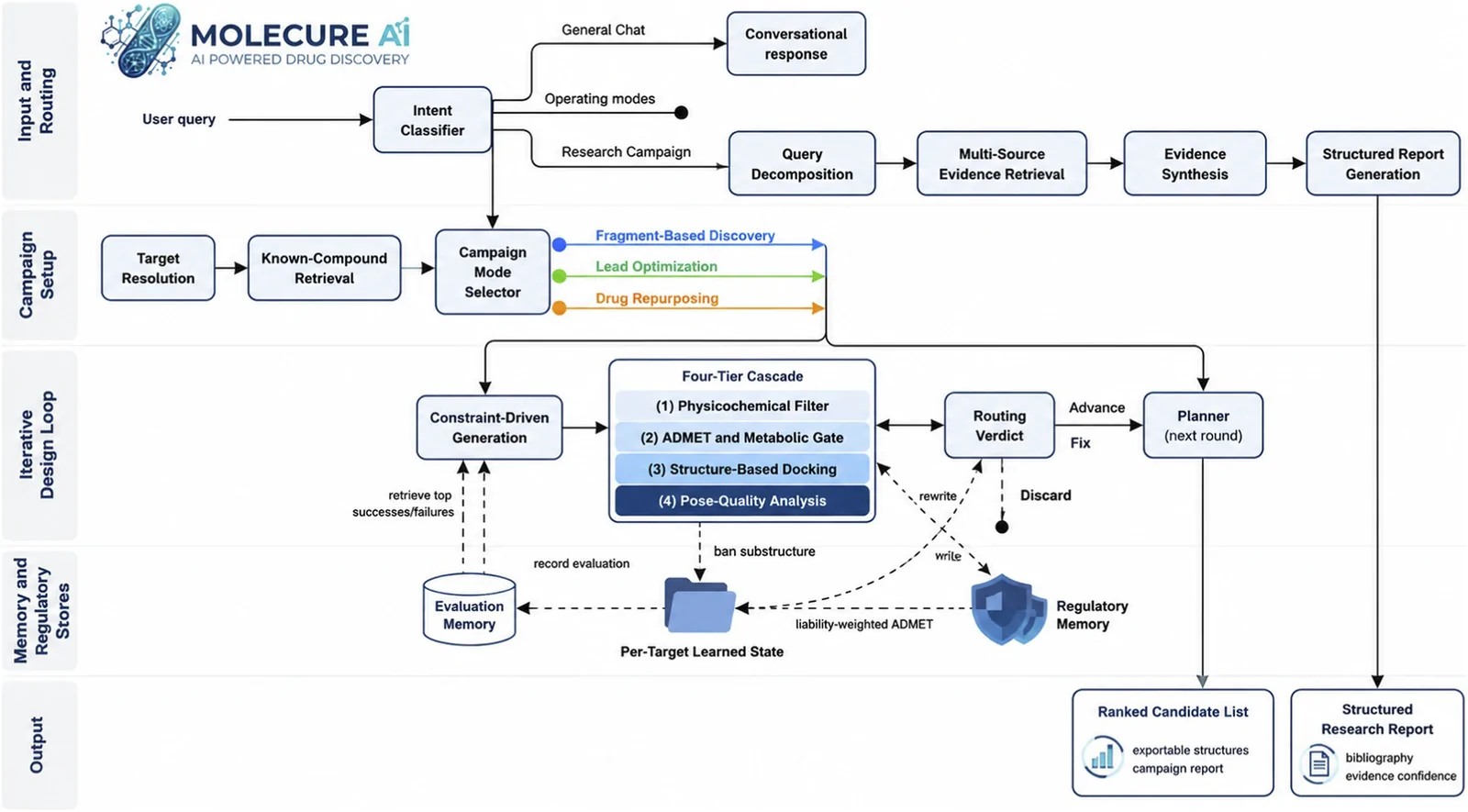
End-to-end workflow of MolecureAI, showing the single-pass routing classifier, the three operating modes (General Chat, Research Campaign, and Generation Campaign), the cost-ordered four-tier evaluation cascade, and the three persistent stores (Evaluation Memory, Per-Target Learned State, and Regulatory Memory) read and written throughout each session.

The first layer, Input and Routing, receives a natural-language query and commits it immediately and irrevocably to one of three operating modes *via* a single-pass intent classifier: General Chat, Research Campaign, or Generation Campaign. No tool is called and no memory is touched until this commitment is made, and it cannot be revised once issued.

The second layer, Campaign Setup, applies exclusively to Generation Campaigns. It performs target resolution, retrieves known bioactive compounds from ChEMBL (Mendez et al., 2019) and PubChem (Kim et al., 2023), and passes the assembled evidence to an autonomous Campaign Mode Selector that commits to one of three design strategies: fragment-based discovery, lead optimization, or drug repurposing. Critically, the strategy is not a user-supplied parameter; it is computed from the evidence before any molecule is generated.

The third layer, Iterative Design Loop, is the computational core of a generation campaign. Constraint-driven generation produces candidate Simplified Molecular Input Line Entry System (SMILES) strings that are immediately evaluated through a four-tier cost-ordered cascade: physicochemical filtering (sub-second), ADMET and metabolic safety gating (seconds), structure-based docking (tens of seconds), and pose-quality analysis (seconds). A Routing Verdict then issues one of three decisions per molecule, Advance, Fix, or Discard, and a Planner either advances the surviving set to the next-generation round or terminates the campaign when convergence or budget criteria are met.

The fourth layer, Memory and Regulatory Stores, operates in parallel with the design loop rather than after it. Three persistent stores are read from and written to throughout each session: Evaluation Memory, which records every assessed structure and its outcome indexed by Morgan fingerprint and learned ChemBERTa embedding; Per-Target Learned State, which accumulates success and failure patterns together with the growing list of banned substructures across all campaigns on the same target; and Regulatory Memory, which holds FDA safety signals, clinical-trial outcomes, and patent-landscape priors with an explicit freshness window. Reads from these stores shape each new design round; writes occur when a routing decision is issued, so that every lesson is available immediately in the following round.

The fifth layer, Output, delivers one of two products depending on the operating mode: a ranked, exportable candidate list with per-molecule property annotations for a generation campaign, or a bibliography-backed structured evidence report for a research campaign.

All architectural invariants that enforce correctness across these layers are the same: every scoring weight renormalizes to one when a component is unavailable; every structure is canonicalized before evaluation; every routing verdict is recorded immutably; and no expensive computation is invoked before the preceding cheaper gate is cleared. Figure 2 details the two-layer MCP tool server that underpins every external data access across all modes. Implementation details deferred from the main text, including scoring weight tables, metabolic profiling parameters, and docking preparation protocols, are provided in the Supplementary Material (Supplementary Sections S1, S2, S8, and S13). The subsections below specify each layer and its internal logic in turn (Supplementary Sections 2.1–2.9), followed by the experimental setup (Supplementary Section 2. 10).

**FIGURE 2:**
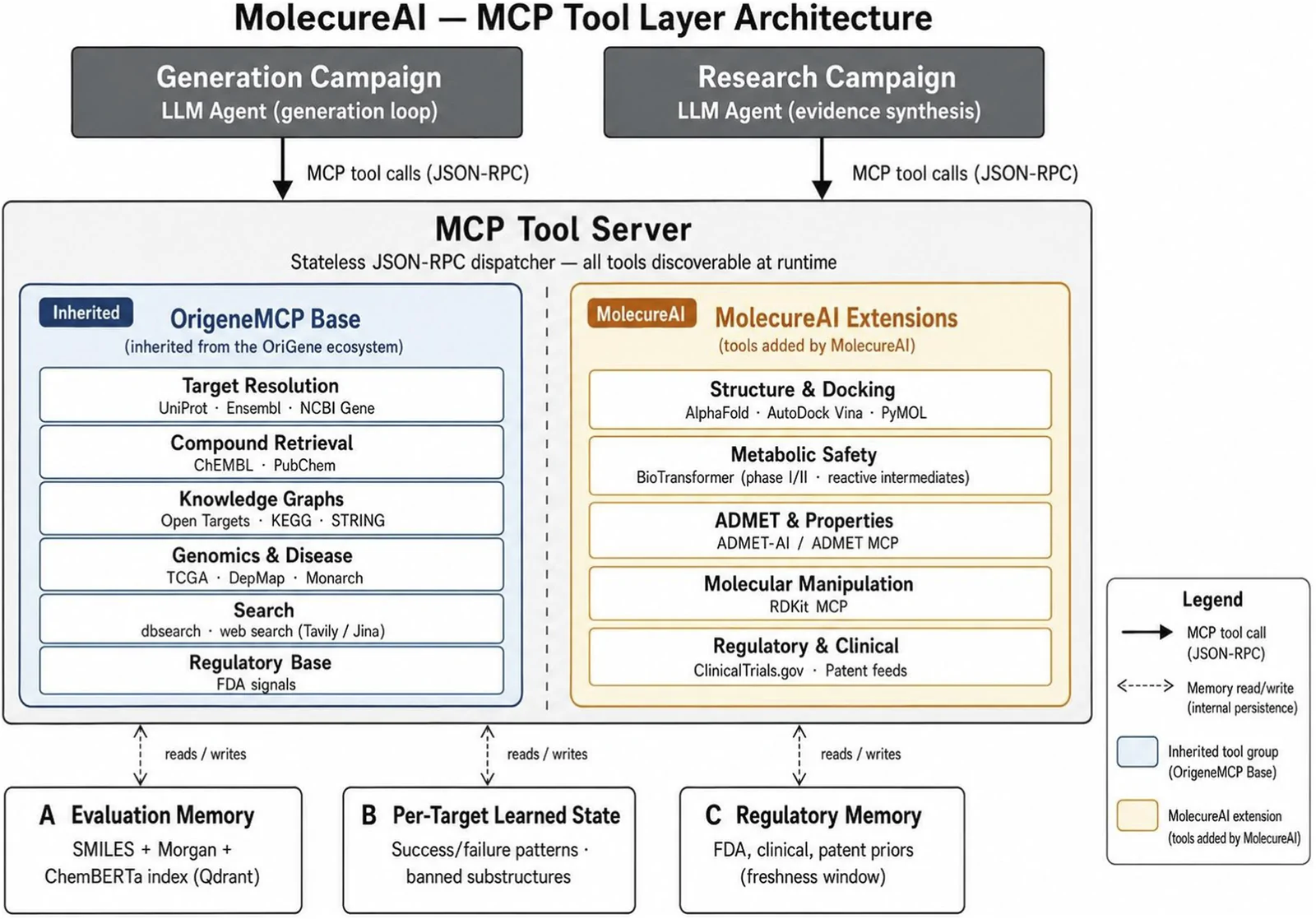
Two-layer MCP tool server underpinning all external data access in MolecureAI. The OrigeneMCP Base layer (inherited from the OriGene ecosystem) supplies target resolution, compound retrieval, biomedical knowledge graphs, and regulatory signals; the MolecureAI Extensions layer adds structure and docking tools, metabolic safety profiling, ADMET prediction, and extended clinical intelligence. Dashed arrows indicate reads from and writes to the three persistent stores.

Each of the five layers is designed to degrade gracefully rather than halt when a dependency is unavailable. Unreachable services are skipped or replaced with neutral default values, with the corresponding scoring weight redistributed or absorbed accordingly, while memory retrieval falls back to a stateless context when the vector store cannot be reached. In every case the cascade continues, the routing verdict is issued with reduced-confidence metadata, and the degraded state is recorded immutably so that downstream components and the final report can distinguish evaluated tiers from skipped ones.

### 2.1 Session routing and target resolution

All scientific and computational work in MolecureAI is gated by a single-pass intent classifier that reads the natural-language query and commits the session to one of three operating modes before any MCP tool call is issued or any memory is touched. This commitment is made first and made once: the three operating modes are mutually exclusive, their assignment is recorded immutably, and no subsequent component can revise it. The immutability is a deliberate correctness guarantee, it prevents a session from silently shifting from a targeted generation campaign to an unconstrained chat response partway through execution.

General Chat terminates immediately with a response drawn from the LLM’s parametric knowledge. No MCP tool is called, no memory is written, and no scientific claim is traceable. This mode exists for usability and is explicitly excluded from all scientific claims made in this paper.

Research Campaign and Generation Campaign both consume MCP tool calls. The classifier assigns these modes by reading three evidence classes in the query: the presence of a named or described molecular target, the presence of generative-intent keywords (design, optimize, find candidates), and the presence of repurposing keywords. A query with a target but no generative intent is routed to Research; a query with both is routed to Generation. The routing decision is recorded immutably for audit.

For both research and generation campaigns, the session proceeds through target resolution once the mode is committed. Target resolution converts the query’s target description into a canonical machine-readable record using the OrigeneMCP Base tools: UniProt (UniProt Consortium, 2023) for protein identity and gene-name disambiguation, Ensembl (Harrison et al., 2024) and NCBI Gene (Sayers et al., 2022) for genomic context, and Open Targets (Ochoa et al., 2023) for target-disease association evidence ranked by genetic evidence score. The resolved record contains the canonical UniProt accession, human gene symbol, target class (kinase, G protein-coupled receptor [GPCR], nuclear receptor, protease, protein-protein interaction [PPI] interface, or other), and the top disease associations.

Structure resolution follows and is required for the docking tier in a generation campaign. The Protein Data Bank (Berman et al., 2000) is queried for experimentally determined structures; co-crystal structures with a small-molecule ligand and resolution below 2.5 Å are preferred. If no acceptable experimental structure is available, AlphaFold2 (Jumper et al., 2021) generates a predicted model; a low-confidence flag is propagated to the routing module so that docking-score uncertainty is reflected in the composite score weight. The resolved structure and pocket center of mass are cached in the session record and reused across rounds.

Known-compound retrieval then assembles the seed set that feeds the strategy selector. ChEMBL (Mendez et al., 2019) is queried for bioactivity records against the resolved target, filtered to curated ${\text{K}}_{i}$ , ${\text{K}}_{d}$ , ${\text{IC}}_{50}$ , and ${EC}_{50}$ measurements with explicit numeric values; units are normalized to nM and inequality records are excluded. PubChem (Kim et al., 2023) bioassay data supplements ChEMBL for targets with sparse coverage. Each retrieved structure is canonicalized with RDKit (Landrum, 2006), deduplicated by InChIKey (a hashed chemical identifier), and passed through a quality gate that filters pan-assay interference compounds (PAINS), scores scaffold diversity (core-framework structural variety), and selects the optimal seed subset for the strategy selector. To mitigate the risk of re-generating known actives, the system maintains a dynamically built blocklist of known inhibitors for each target, assembled from ChEMBL bioactivity records, FDA-approved drugs, and a set of hardcoded reference inhibitors (e.g., gefitinib and erlotinib for EGFR). During candidate evaluation, each generated structure is checked against this blocklist *via* exact InChIKey match, canonical SMILES match, and Bemis-Murcko scaffold match (Bemis and Murcko, 1996); any candidate whose scaffold is already represented in the known active space is flagged accordingly in its novelty report. The blocklist’s coverage is bounded by the structural-data completeness of each source API; a *post hoc* analysis of coverage gaps is reported in Supplementary Section 13. An independent inter-round diversity gate additionally enforces a minimum pairwise Tanimoto similarity distance of 0.40 between generated molecules to prevent structural collapse within a campaign.

### 2.2 Evidence-driven campaign strategy selection

A central design decision in MolecureAI is that the campaign strategy is not a hyperparameter the user supplies, it is computed from the evidence. Before a single generative step is attempted, the system autonomously commits to one of three design strategies: fragment-based discovery (FBDD), lead optimization, or drug repurposing. The selection is performed by a stateless decider that reads five orthogonal signals extracted from the seed compound set and the resolved target context, maps each signal to a score in [−1,+1] under the convention that negative values favor fragment-space exploration and positive values favor lead optimization, and combines them into a composite score $\Phi =\sum_{k}w_{k}s_{k}$ whose weights reflect medicinal-chemistry priority. The rationale for this architecture is straightforward: a researcher who does not know which strategy is appropriate, the very situation in which a computational assistant is most valuable, cannot supply a correct hyperparameter, and a system that defaults silently to one strategy regardless of target context is providing false confidence. Table 3 specifies each signal and its rationale.

**TABLE 3:** Evidence signals for autonomous strategy selection. Each signal returns a score in [−1,+1] (negative → FBDD, positive → lead optimization). Weights sum to 1.0 by construction.

Signal	Weight	What it measures and why it matters
Seed profile	0.30	Fraction of retrieved actives that are Ro3-compliant (fragment-like) *versus* Ro5-compliant (drug-like). A predominantly fragment-like pool means there is room to grow; a drug-like pool means optimization.
Activity density	0.25	Fraction of actives with ${\text{IC}}_{50}<100$ nM after unit normalization. Dense potent data supports SAR-driven optimization; sparse data argues for exploration.
Ligand efficiency	0.20	Drug-likeness per heavy atom, a proxy for how much potency the existing matter buys per unit of size. High efficiency on small matter points to fragments.
Target class	0.20	Family inference from gene and annotation. Kinases, GPCRs, and proteases reward optimization; protein-protein interfaces and disordered or cryptic sites reward fragments.
Query intent	0.05	Keyword evidence in the researcher’s text. A deliberately weak tiebreaker only.

The mapping from Φ to a strategy is asymmetric by design. Values above +0.15 select lead optimization and values below −0.15 select FBDD, while the dead zone |Φ|≤0.15 defaults to FBDD rather than splitting the difference. This is a scientific choice, not a tie-breaking convenience. A campaign that opens in fragment space can expand toward larger chemical matter as structural confidence accumulates, whereas one that commits prematurely to optimization narrows its coverage in a way the budget cannot later undo. Confidence is reported as min(|Φ|/0.5, 1) so that downstream components can weight the decision. Two overrides take precedence over the composite score, both for sound reasons. An explicit user declaration of FBDD is honoured outright, and repurposing keywords route the campaign to the repurposing strategy with high confidence, because repurposing changes the candidate universe from generated structures to approved drugs and cannot be inferred from seed physicochemistry alone. The full decision, including the per-signal breakdown and a human-readable rationale, is recorded immutably for audit.

### 2.3 Constraint-driven generation and the planning loop

Molecule generation in MolecureAI is not an unconstrained language-model sampling step followed by *post hoc* filtering. Instead, the design context for each round is assembled from the campaign’s full accumulated state, encoding the active strategy, design anchor, retrieved memory, avoidance constraints, and target-class property targets as explicit structural directives. The generative model is the same reasoning LLM that drives routing arbitration and research synthesis, accessed through a constrained generation call that enforces SMILES validity and canonical form *via* RDKit (Landrum, 2006) post-processing. This design, a single unified model across all roles, is a deliberate choice to demonstrate that architectural invariants, not model specialization, are responsible for the quality and safety properties of the output. Invalid SMILES strings are discarded without entering the evaluation cascade, and a single repair call is issued for Fix-verdict molecules that carry one correctable structural defect.

The design context for each round is assembled from five components: strategy context, design anchor, retrieved memory (fingerprint, ChemBERTa, and scaffold-SAR graph retrieval), avoidance constraints, and property targets; full specification of each component and its source within the system is described in the Supplementary Material (Supplementary Section 2).

The planner manages the transition between rounds. After all routing verdicts are issued, the planner inspects the Advance set and selects the next anchor: the molecule with the highest composite score for lead optimization, the molecule with the best ligand efficiency for FBDD, and the derivative with the highest seed similarity combined with improved predicted affinity for repurposing. The planner then updates the target’s Per-Target Learned State with the new success and failure patterns, appends any newly banned substructures to the avoidance list, and increments the round counter before issuing the next-generation call.

The campaign terminates under any of three conditions: (1) the maximum number of rounds is reached; (2) the top composite score in the Advance set crosses a user-defined excellence threshold; or (3) the Advance set has been empty for two consecutive rounds, indicating that the current scaffold family is exhausted under the active constraints. On termination, the full ranked candidate list, the per-round attrition statistics, and the final Per-Target Learned State are written to persistent storage and returned to the user as a ranked exportable report.

### 2.4 The cost-ordered evaluation cascade

The central resource-management mechanism of MolecureAI is a strictly ordered, four-tier evaluation cascade in which every candidate must clear a tier before it is eligible for the next. Failure at any tier terminates evaluation immediately and routes the molecule back to the arbitration layer with its failure mode recorded. The ordering principle is not a heuristic: it is a correctness invariant. The cheapest, most selective gates execute first, physicochemical filtering in sub-second time, ADMET and metabolic screening in seconds, so that structure-based docking and three-dimensional pose analysis, which dominate computational cost by one to two orders of magnitude, are invested exclusively in molecules that have already been validated by every cheaper criterion. The result is that expensive computation is concentrated where it is epistemically justified rather than uniformly applied.

Tier 1 computes physicochemical descriptors and screens drug-likeness, synthetic accessibility, and pan-assay interference, applying target-class property windows rather than universal cutoffs. It uses a soft-then-hard gate, admitting molecules that exceed a soft bound but stay within a roughly twenty-percent buffer so that borderline matter is not discarded by an arbitrary line, while fragment campaigns enforce the Rule of Three as a hard gate to keep docking budget off non-fragments. Tier 2 profiles ADMET across cardiac, mutagenic, blood-brain-barrier, solubility, and metabolic-inhibition endpoints, with thresholds adapted to the target class. ADMET predictions are produced by ADMET-AI (Swanson et al., 2024) (Python package admet-ai, v1), a Chemprop-RDKit graph neural network (Chemprop v1.6.1) trained on 41 ADMET datasets from the Therapeutics Data Commons (TDC v0.4.1): 10 regression and 31 classification endpoints derived from experimental-assay molecules. The two safety-critical binary classifiers driving CRITICAL discard in this pipeline are hERG cardiotoxicity (binary blocker classification) and AMES mutagenicity (binary Ames test outcome), both sourced from their respective TDC single-task datasets. Single-task models were trained on random 80/10/10 train/validation/test splits across five seeds and ensembled at inference, while the 22-dataset TDC ADMET Benchmark Group subset used for cross-tool benchmarking follows TDC’s Murcko scaffold split. ADMET-AI achieves the highest average rank on the TDC ADMET Benchmark Group leaderboard, with AUROC > 0.85 for 20 of the 31 classification endpoints. The original publication does not report calibration metrics such as Brier score or expected calibration error; predicted probabilities are therefore used as relative risk scores rather than calibrated absolute probabilities. ADMET-AI does not implement applicability-domain criteria; in this pipeline, Tier 1 physicochemical pre-filtering restricts prediction to drug-like chemical space, and CRITICAL discard thresholds (hERG ≥0.90 , AMES ≥0.80 ) are set conservatively to limit sensitivity to out-of-domain predictions. Tier 2.5 is a dedicated metabolic screening step, distinct from the four numbered tiers, that assesses predicted metabolite liabilities before routing, combining parent-molecule and metabolite safety signals into a unified gate (Section 2.5). Tier 3 docks survivors against the resolved structure with AutoDock Vina (Trott and Olson, 2010), and because docking is the dominant cost it runs only after explicit confirmation; declining removes the tier and triggers renormalization of the scoring weights. Tier 4 scores the docked pose on hydrogen-bond contacts, steric clash, and pocket occupancy; when three-dimensional analysis is unavailable it contributes its default neutral value rather than switching to an alternative scoring mode. Candidate attrition per tier is illustrated in Figure 3.

**FIGURE 3:**
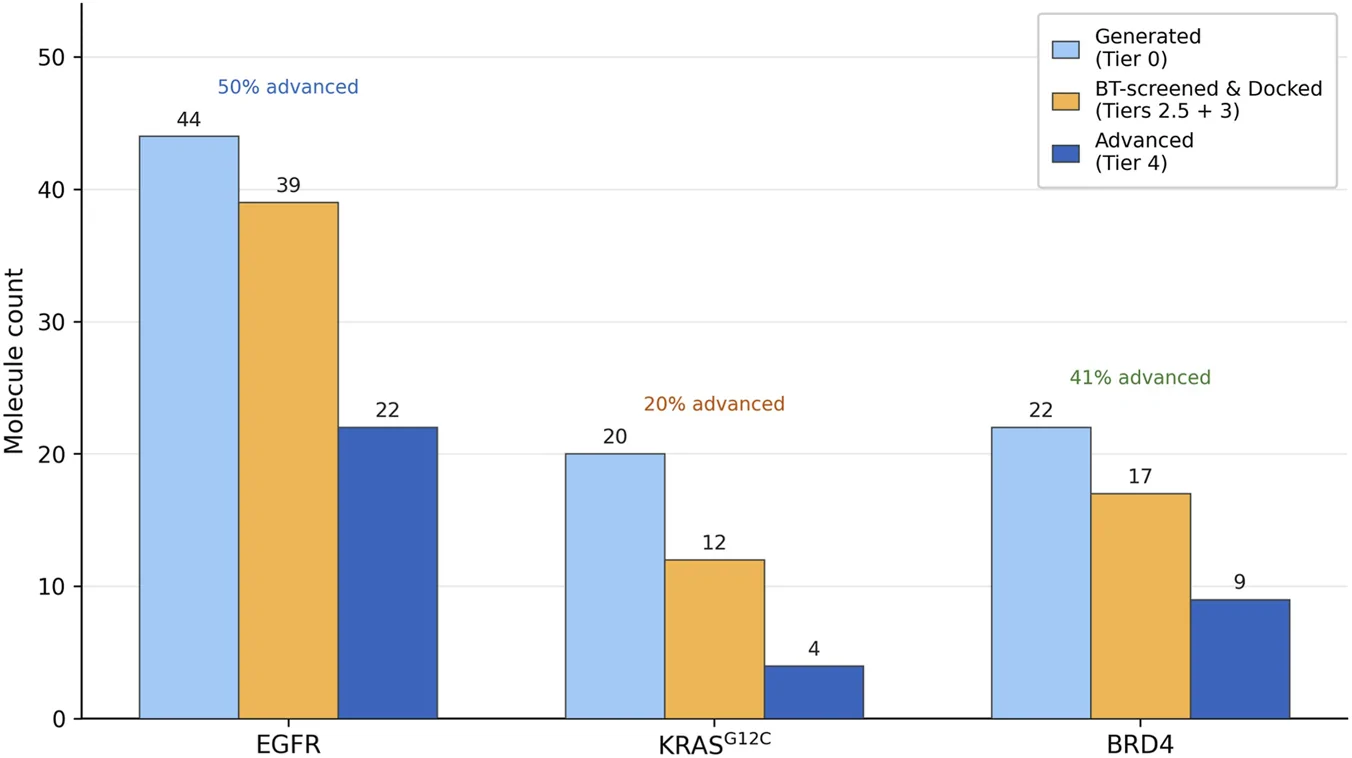
Cascade attrition funnel for EGFR, ${\text{KRAS}}^{\text{G}12\text{C}}$ , and BRD4, showing candidate counts surviving each evaluation tier. No candidate was eliminated at the metabolic gate.

A compensatory composite score prioritizes molecules that survive the cascade. It reweights predicted safety above raw potency, because a potent molecule with a cardiac or mutagenic liability is worth less to a campaign than a slightly weaker clean one. When a target carries documented liabilities such as QT or hepatotoxicity signals, an independent regulatory liability term (weight 0.07) in the composite score penalizes molecules whose predicted cardiac or metabolic profiles match the historical class pattern, steering design toward safer chemotypes without altering the core ADMET weight. The full component weight breakdown and advancement thresholds are detailed in Table 4.

**TABLE 4:** Key operational parameters of the evaluation cascade and routing layer. Default values apply unless overridden at session initialization.

Parameter	Default	Role
Tier 1 soft-buffer margin	20%	Admits borderline matter before hard cutoff.
hERG WARNING threshold	0.50	Cardiac signal above which a graded composite penalty is applied.
hERG CRITICAL threshold	0.90	Cardiac signal above which molecule is blocked before docking.
Ames mutagenicity test (AMES) WARNING threshold	0.50	Mutagenic signal above which a graded composite penalty is applied.
AMES CRITICAL threshold	0.80	Mutagenic signal above which molecule is discarded and substructure banned.
Routing confidence band	± 0.10	Probability band around any threshold within which LLM arbitration is invoked instead of deterministic discard.
Composite scoring weights (sum to 1.0)		
ADMET safety	0.23	Contribution of predicted safety endpoints to total score.
QED	0.20	Contribution of physicochemical drug-likeness.
Structural	0.19	Contribution of steric clash and binding geometry penalties.
Docking	0.17	Contribution of AutoDock Vina predicted binding affinity.
Solubility	0.09	Contribution of predicted aqueous solubility.
Regulatory	0.07	Penalty for matching known target-class liabilities.
SA score	0.05	Contribution of synthetic accessibility.
Advancement thresholds^a^		
Minimum composite score	>0.59	Nominal threshold for overall viability.
Maximum docking score	≤−7.0	Nominal computational hit-filter threshold (kcal/mol), calibrated on deep enzymatic pockets.
Critical ADMet alerts	0	Candidate must pass all hERG and AMES critical gates.
Memory retrieval α/β/γ	0.50/0.35/0.15	Performance/similarity/recency balance in evaluation memory.
Recency decay window	10 rounds	Linear decay to zero over ten prior rounds.

^a^ Composite score and docking thresholds are target-agnostic nominal defaults, calibrated on deep enzymatic pockets (kinase ATP sites, Switch-II allosteric pockets). Shallow, solvent-exposed binding surfaces such as the BRD4 BD1 acetyl-lysine recognition site systematically yield weaker Vina scores at equivalent binding quality; for such targets the routing layer’s arbitration (Section 3, and see the BRD4 discussion in Section 3.1) admits candidates below the nominal docking and composite cutoffs, as reflected in the BRD4 candidate scores reported in Supplementary Section 13.

Routing is the single arbitration layer for a molecule’s fate, and it operates in two stages to conserve reasoning budget. A deterministic filter applies priority-ordered pharmacological rules, and the first rule that fires decides the outcome with no model call. Mutagenic or reactive matter is discarded outright, a scaffold already banned in this or a prior campaign is discarded as a known waste of budget, and a single repairable property violation is marked for correction next round. Only when the deterministic filter is silent, or when a critical signal carries low predictive confidence (defined as a predicted probability within 0.10 of the relevant threshold, e.g. human Ether-à-go-go Related Gene (hERG) probability between 0.40 and 0.60 when the threshold is 0.50), is the full property profile handed to a reasoning model for arbitration grounded in the target class and the campaign’s accumulated success and failure patterns. The verdict, advance, fix, or discard, then drives every downstream decision without being second-guessed.

Table 4 summarizes the key operational parameters of the cascade and routing layer. All thresholds are defaults that can be overridden by an explicit user instruction at session initialization; overrides are recorded immutably alongside the routing verdicts they affect.

### 2.5 Metabolic safety as an in-loop gate

A central safety architecture of MolecureAI is the positioning of metabolic and cardiac liability assessment *inside* the generation loop, not as a *post hoc* filter applied to a finalized candidate list. A candidate bearing a critical hERG signal, for example, is blocked before any docking call is issued, and the responsible substructure is written immediately to the target’s persistent Per-Target Learned State as a banned pattern for all subsequent rounds (Section 2.3, component iv). Metabolic profiling is performed by BioTransformer (Djoumbou-Feunang et al., 2019), a rule- and machine-learning hybrid that predicts Phase I and Phase II metabolites, reactive intermediates, and gut-microbiota biotransformations. Profiling is applied during the generation cascade to every candidate that clears Tier 1 and in the repurposing pipeline to the source drug before any derivative is proposed (Section 2.9). In both cases the predicted liabilities are recorded alongside the parent structure so that a reactive intermediate flagged in one round is suppressed thereafter rather than re-proposed and re-evaluated at full cost.

### 2.6 Persistent, structurally indexed memory

One of the most consequential architectural gaps in existing autonomous drug design systems is that chemical knowledge accumulated during a campaign does not survive its end: the next campaign on the same target begins from a blank prior regardless of how much was learned before. MolecureAI addresses this gap through three distinct persistent memory stores, each designed to answer a qualitatively different retrieval question. The separation by type is intentional, collapsing all memory into a single store would require a single retrieval strategy that cannot simultaneously optimize for precise analogical recall, cross-campaign structural accumulation, and regulatory freshness.

Evaluation Memory is the per-molecule store. Every assessed structure is recorded with its scaffold, routing verdict, composite score, stage of failure, and an annotated reason. Records are deduplicated by canonical SMILES per target, with bounded capacity so that older evaluations are retired to make room for new ones. Retrieval ranks records by a composite score that balances historical performance, structural proximity to the current design anchor, and recency,

$$r\left(n\right)=\alpha \text{⋅}perf\left(n\right)+\beta \text{⋅}T\left(n,a\right)+\gamma \text{⋅}rec\left(n\right),\alpha =0.50,\;\beta =0.35,\;\gamma =0.15,$$

where T(n,a) is Morgan-fingerprint Tanimoto similarity to anchor a and the recency term decays linearly over ten rounds. When no anchor exists (first round of a new campaign), the similarity weight β folds into the performance weight so that $\alpha^{\prime }=\alpha +\beta =0.85$ and $\gamma^{\prime }=\gamma =0.15$ , ensuring that retrieval returns the historically strongest matter rather than defaulting to an arbitrary nearest-neighbour. When Evaluation Memory is empty, no prior examples are available to inform the design, and generation proceeds from target class priors alone.

Three retrieval modes operate over the evaluation store. *Fingerprint search* gives precise recall of close analogues with known activity, which is what a lead-optimization round needs. A *semantic index* built on ChemBERTa (Chithrananda et al., 2020) 768-dimensional dense embeddings recovers compounds that are pharmacologically related but structurally distant: two molecules with the same binding pharmacophore but different scaffolds will be close neighbours in ChemBERTa embedding space even when their Tanimoto similarity is negligible. This mode is used during scaffold-hopping campaigns. A *scaffold-SAR graph* traverses scaffold families and activity relationships between evaluated molecules, recording scaffold similarities, target outcomes, and potency cliffs as traversable edges. This mode detects scaffold-family exhaustion and suggests scaffold hops.

Per-Target Learned State accumulates bounded lists of success and failure patterns, a growing set of banned substructures, and scaffold outcome histories across campaigns on the same target. These patterns carry forward as structural constraints, so that the system does not repeat previously failed chemotypes.

Regulatory Memory stores FDA safety signals, clinical-trial outcome summaries, and patent-landscape information organized by target identity and protein class, with finite temporal validity so that safety intelligence is refreshed periodically rather than accumulated indefinitely without revision. These priors participate in the composite scoring function through an independent regulatory liability term (weight 0.07) drawn from hepatotoxicity and metabolic inhibition predictions and compared against the stored historical priors. A molecule whose predicted hepatotoxicity profile matches a documented FDA boxed warning for the target class receives a penalized regulatory score, steering design toward safer chemotypes without altering the core ADMET weight.

### 2.7 Regulatory intelligence as a design prior

In most computational drug discovery pipelines, regulatory and clinical context arrives at the end of a campaign as a compliance layer, examined after candidates have been ranked, not before they were designed. MolecureAI inverts this order: regulatory and clinical evidence is retrieved during session initialization and encoded as a design prior that actively shapes generation constraints before any molecule is proposed. During initialization, the system queries FDA safety signals, clinical-trial outcome summaries, and patent-landscape information for the target and its class. These priors carry direct computational consequences: a target with documented cardiac or hepatic liability causes the regulatory liability term in the composite scoring function to penalize molecules whose predicted drug-induced liver injury (DILI) or CYP inhibition profiles match the historical class pattern, so that the system designs more conservatively against precisely the liability class the target has already exhibited in the clinic.

### 2.8 Research campaign: evidence synthesis without generation

Not every scientific question about a target requires generating molecules, and not every researcher who approaches a new target is ready to commit to a generation campaign. MolecureAI addresses this through a dedicated research campaign mode that answers scientific questions through structured, multi-source evidence retrieval and synthesis, without entering the iterative design loop or producing any candidate structures. For the researcher, it provides a bibliography-backed landscape of the target, including known inhibitor chemotypes, clinical-trial outcomes, resistance mechanisms, and IP context, organized as a structured report before any computational chemistry budget is spent.

When the intent classifier routes a query to the research mode, the system decomposes it into a set of evidence subquestions,for instance the known inhibitor landscape, clinical-trial outcomes, resistance mechanisms, and comparative toxicity profiles for a given target. Each subquestion is dispatched to the most appropriate external source: web-search engines for recent literature, ChEMBL (Mendez et al., 2019) for bioactivity, PubChem (Kim et al., 2023) for compound data, Open Targets (Ochoa et al., 2023) for target-disease associations, and ClinicalTrials.gov (ClinicalTrials.gov, 2024) for trial outcomes. Calls are executed concurrently where the sources are independent, and the returned unstructured text is parsed into structured evidence entries with traceable bibliographic metadata.

The retrieved evidence is assembled into a structured report whose sections are determined by the query type: target biology, known actives, clinical precedent, IP landscape, and a synthesis narrative that highlights convergent findings and open questions. The report is bibliography-backed in the same sense as a systematic review: every factual claim carries a traceable data source. Because research and generation campaigns query the same external databases through the shared MCP tool layer, a researcher can transition from literature review to structure generation on the same target without redefining the data sources; each campaign evaluates evidence independently. Detailed query decomposition rules and source-to-section mappings are provided in the Supplementary Material (Supplementary Section 2).

### 2.9 Drug repurposing as an autonomous campaign mode

Drug repurposing, the systematic identification of new therapeutic indications for approved or clinically advanced compounds, offers a well-documented development advantage: the repurposed molecule enters the pipeline with an existing human safety record, characterized pharmacokinetics, and a regulatory precedent that substantially reduces time to clinical evaluation (Pushpakom et al., 2019; Himmelstein et al., 2017). In MolecureAI, repurposing is one of three autonomous campaign modes selected before generation begins, alongside fragment-based discovery and lead optimization (Section 2.2), triggered by explicit repurposing intent in the user’s query detected through keyword matching. Unlike the other two modes, which are inferred from seed physicochemistry and target class, repurposing originates directly from the stated therapeutic intent.

The user names the source drug explicitly; the system resolves its canonical SMILES through a fallback chain of extraction, parsing, compound lookup, and inference. Before generation begins, the source drug undergoes a structured safety audit: ChEMBL supplies regulatory status and approved indication; FDA queries retrieve post-market safety signals and contraindications; OpenTargets maps the new target to its associated diseases; ADMET profiling identifies off-target liabilities such as hERG or CYP inhibition; and BioTransformer (Djoumbou-Feunang et al., 2019) predicts metabolite liabilities, flagging reactive classes (quinone, epoxide, Michael acceptor). A contraindication gate cross-references the retrieved adverse-event alerts against the new indication; if a safety conflict is detected, the campaign aborts immediately with a structured report.

If the source drug passes audit, its SMILES becomes the initial seed for the standard molecular design loop. The full repurposing context (source identity, original and new indications, new target gene, FDA status, known off-targets, and metabolite liabilities) is encoded as explicit constraints, so that derivatives preserve the pharmacologically validated scaffold while avoiding the source’s liability classes.

### 2.10 Experimental setup and evaluation protocol

The empirical evaluation comprised four sequential arms, each isolating a distinct architectural claim while drawing on the evidence from the preceding one. Experiments 1 to 3 held the generator constant at groq-8b to ensure that observed behaviors reflect pipeline-level architecture rather than model-specific capability; the LLM backbone benchmark then varied the generator to test whether those properties hold independently of model choice. Detailed protocols for all experimental arms are provided in the Supplementary Material (Supplementary Sections 5–8).

Table 2 states the protocol basis and current evidentiary status for the architectural contributions and core claims introduced in Section 1. This taxonomy clearly separates experimentally validated components from those that are architecturally implemented but reserved for future benchmarking. Quantitative outcomes for all validated claims are reported in Section 3 and Section 4.

Experiment 1 asks whether the full pipeline operates as a coherent system across structurally diverse targets. Three benchmark targets were selected to span target-class diversity: EGFR (kinase, dense ChEMBL coverage), ${\text{KRAS}}^{\text{G}12\text{C}}$ (GTPase, cryptic allosteric pocket, eight heterogeneous ChEMBL seeds), and BRD4 (bromodomain, moderate coverage). One campaign was executed per target with twenty iterations. The pipeline employed a graded penalty routing system: moderate predicted liabilities (hERG 0.50 to 0.89, AMES 0.50–0.79) penalized the composite score but did not block advancement if offset by strong binding affinity; only hERG ≥0.90 or AMES ≥0.80 triggered CRITICAL discard. BioTransformer metabolic profiling was applied to all candidates clearing Tier 1 to 2. Key metrics reported are campaign totals, per-tier attrition, wall-clock timing, scaffold recovery, and advance rates per target; outcomes are reported in Section 3.1.

Experiment 2 asks which pipeline component produces the observed behavior. Because Experiment 1 activates all components simultaneously, it cannot attribute performance to any single one. A systematic five-condition ablation across all three targets therefore selectively disables individual components to measure their independent and joint contributions. The five conditions form an additive sequence in which each subsequent condition introduces one additional pipeline tier to the bare LLM baseline (Condition A). Conditions B through D were implemented *via* conditional deactivation of individual tiers to ensure identical code paths, eliminating implementation confounds. All conditions were evaluated *post hoc* by a unified independent evaluator computing seven metrics: SMILES validity, drug-likeness (QED), synthetic accessibility (SA score), ADMET pass rate (44 endpoints), mean AutoDock Vina docking score, percentage of candidates achieving ≤−7.0 kcal/mol (the hit threshold), and Bemis-Murcko scaffold diversity.

Experiment 3 asks what CampaignMemory specifically contributes. Condition E in Experiment 2 still does not isolate memory from the other active tiers, so a dedicated ablation on EGFR held all components constant and varied only the Qdrant-backed memory store across three rounds of 20 iterations each ( approx90 molecules per condition).

#### 2.10.1 LLM backbone benchmark

Experiments 1 to 3 leave open whether the validated properties are architecture-level invariants or artifacts of the groq-8b generator. Four backends were evaluated on EGFR (groq-8b, claude-sonnet, gpt-4o, and gemini-1.5-pro), with all cascade thresholds, docking protocol, and evaluation metrics held constant; only the generator varied. The benchmark applied the strict *post hoc* criteria: composite score >0.59 , docking ≤−7.0 kcal/mol, and zero critical ADMET alerts (hERG ≥0.90 or AMES ≥0.80 ; see Supplementary Material, Supplementary Section 9).

For contextual comparison against the closest available LLM-agent baseline, ChemCrow (Bran et al., 2024) was executed under its default operating configuration using GPT-4o to generate 30 EGFR candidate molecules in a single-shot prompt. All ChemCrow-generated molecules were subsequently evaluated using the same ADMET and docking pipeline applied to MolecureAI candidates in Experiment 1 (Section 2.4), ensuring that the downstream evaluation, though not the generation protocol, was held identical between the two systems. This comparison is reported in Table 1, Panel B, alongside the architectural positioning in Panel A. The Llama 3.1 8B (Groq) campaign underlying this comparison (Table 1, Panel B) is an independent run from the Llama 3.1 8B row in the four-backend benchmark reported below (Table 5); the two use different iteration budgets (27 vs. 20) and serve distinct purposes, contextual baseline comparison *versus* controlled backend recalibration.

**TABLE 5:** LLM backend benchmarking results on EGFR (20 iterations per campaign). Inference time and estimated API cost reflect the actual 20-iteration campaigns.

Backend	Tier-3 evaluated	Objective advances	Discarded	Advancement rate	Inference Time (min)	Est. API cost (USD)
Claude 4 sonnet	49	40	9	81.6%	117.6	$3.67
Llama 3.1 8B (groq)	27	25	2	92.6%	40.1	$0.01
GPT-4o (OpenAI)	29	23	6	79.3%	65.4	$2.27
Gemini pro Latest	23	19	4	82.6%	38.0	$0.94
Total	128	107	21	83.6%	N/A	N/A

Autonomous strategy selection and research campaign synthesis have no dedicated experimental arm in the present study; Table 2 states their current evidentiary status, and both constitute priority objectives for the next evaluation cycle (Section 4.8).

## 3 Results

As summarized in Table 2, this section focuses exclusively on the experimentally validated contributions of the MolecureAI pipeline. Accordingly, the following results are organized around the empirical contributions described in Section 2.10. Each subsection presents the quantitative outcomes for the corresponding arm; the Discussion (Section 4) interprets their combined implications.

### 3.1 Experiment 1: end-to-end cascade validation across three structurally diverse targets

One campaign of exactly 20 iterations was executed per target with the generator held constant. Aggregate statistics are summarized in Table 6.

**TABLE 6:** Aggregate campaign statistics for Experiment 1 (three targets, 20 iterations each). Pre-docking survivors cleared Tiers 1 to 2; strict ADMET pass = zero critical alerts. Throughput in mol $h^{-1}$ .

Target	Generated	Pre-docking survivors	Strict ADMET pass	Advanced	Time (s/h)	Throughput
EGFR	44	39	15	22	4,978/1.38	32
KRA $S^{\text{G}12\text{C}}$	20	12	5	4	4,620/1.28	16
BRD4	22	17	8	9	2,660/0.74	30
Total	86	68	28	35	12,258/3.40	25

The language model produced 86 SMILES strings; 68 (79.1%) entered the combined Tier 2.5 to Tier 3 stage after Tier 1 and Tier 2 filtering. Of these, 35 received a routing verdict of advance (51.5% of those docked, 40.7% of total generated). For EGFR, 39 candidates passed the pre-docking tiers, yet only 22 passed all four tiers. Advance rates were 50.0% for EGFR (22/44), 20.0% for ${\text{KRAS}}^{\text{G}12\text{C}}$ (4/20), and 40.9% for BRD4 (9/22).

The attrition pattern is broken down by target in Figure 3. The step-down from Generated to Advanced reflects the cumulative effect of all four tiers. The pre-docking and Docked populations coincide because no candidate was eliminated between Tier 2.5 and Tier 3; BioTransformer detected no risky metabolites. Of the 86 generated candidates, 18 (20.9%) were removed before docking, concentrating the dominant computational cost on the 68 survivors.

Figure 4 locates the 35 advanced candidates in the QED *versus* binding affinity and MW and composite score planes. Docking scores and ADMET predictions establish that the cascade advanced candidates with plausible drug-like profiles; they do not constitute evidence of biochemical activity (see Section 4.7 for a discussion of docking’s limitations as a screening proxy). Mean synthetic accessibility (SA) scores for the full generated sets were 3.03 (EGFR), 3.61 ( ${\text{KRAS}}^{\text{G}12\text{C}}$ ), and 3.00 (BRD4), indicating that the majority of proposed structures fall within a synthetically tractable range.

**FIGURE 4:**
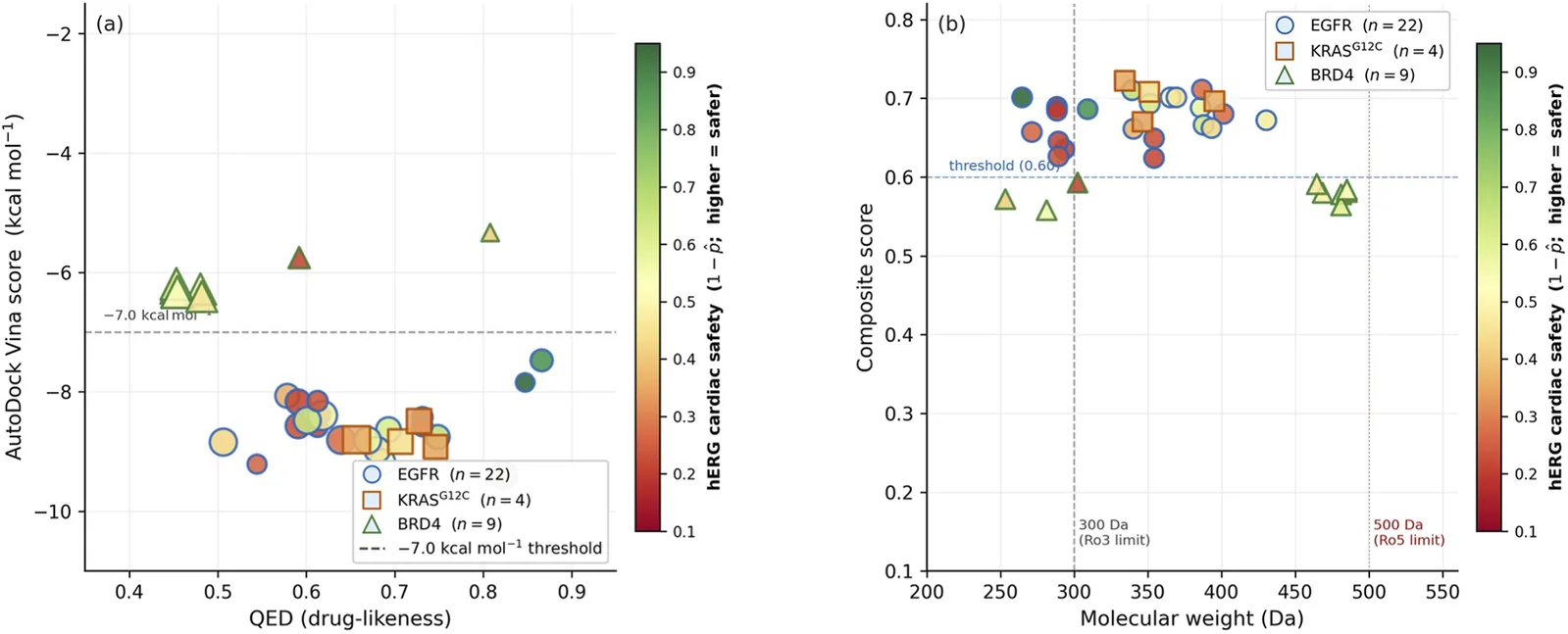
Property portrait of the 35 advanced candidates across the three campaigns. **(a)** QED (drug-likeness) versus AutoDock Vina binding affinity, with the –7.0 kcal mol^-1^ hit-filter threshold shown as a dashed line. **(b)** Molecular weight versus composite score, with the composite-score threshold (0.6) and the Lipinski/Rule-of-Five molecular-weight guidelines (300 Da and 500 Da) shown as dashed lines. Marker shape indicates target (circle: EGFR; square: KRASG12C; triangle: BRD4); colour indicates predicted hERG cardiac safety (green = safer, red = higher risk).

#### 3.1.1 EGFR

All 22 advanced candidates satisfied the Lipinski Rule of Five (MW 261–429 Da, mean 339 Da; QED 0.51 to 0.87, mean 0.67) (Lipinski et al., 1997; Bickerton et al., 2012). Docking scores ranged from −7.47 to −9.45 kcal ${\text{mol}}^{-1}$ (mean −8.61 kcal ${\text{mol}}^{-1}$ ), all below the −7.0 kcal ${\text{mol}}^{-1}$ computational hit-filter threshold (Fukunishi et al., 2018). Mean predicted hERG inhibition was 0.583 (range 0.047–0.814; lower is safer), within the range the same predictive model assigns to approved kinase inhibitors such as erlotinib (predicted hERG ≈0.6 ) (Finlay and Ward, 2017; Krizovska et al., 2013); all values remained below the critical blocking threshold of 0.90. Mean composite score was 0.67 (range 0.624–0.711). Without explicit chemotype instruction in the generation prompt, the campaign computationally converged toward two dominant scaffold families (Bemis-Murcko scaffold IDs, Supplementary Table): a triazol-3(4H)-one-aminopyrimidine series bearing an acrylamide (Michael-acceptor) piperidine-ether substituent (scaffold S3, 8/22 candidates, 36%), structurally reminiscent of the covalent-warhead strategy used by third-generation EGFR inhibitors such as osimertinib, and a 4-anilinoquinazoline series (scaffold S1, 5/22 candidates, 23%) related to the chemotype of first-generation reversible inhibitors such as erlotinib and gefitinib. The remaining eleven candidates were distributed across seven minor scaffolds (S2, S4-S9; 1–2 candidates each). This convergence is consistent with cascade steerability toward known chemical space, but does not on its own constitute biochemical validation of the generated candidates, or evidence of pharmacological activity, or establish that the design strategy achieves genuine autonomous exploration rather than reproduction of well-represented pre-training chemotypes (see Section 4.3 for the corresponding training-data-depth analysis). During candidate evaluation, the pipeline’s internal novelty gate explicitly flagged the scaffold proximity of these structures to known actives, reflecting the high density of the explored EGFR chemical space.

#### 3.1.2 KRA $S^{\text{G}12\text{C}}$

The four advanced candidates (MW 334–395 Da, mean 357 Da) achieved docking scores of −8.49 to −8.92 kcal ${\text{mol}}^{-1}$ (mean −8.76 kcal ${\text{mol}}^{-1}$ ) against the Switch-II allosteric pocket (PDB 6N2J) (Fell et al., 2018), all exceeding the computational hit-filter threshold. Mean composite score was 0.70. Predicted hERG was 0.625 (range 0.554–0.654). All four candidates shared a single Bemis-Murcko scaffold class (diversity ratio 0.25) (Bemis and Murcko, 1996; Zhang et al., 2019), consistent with early convergence on the geometrically restricted Switch-II cavity (Zhu et al., 2022). ${\text{KRAS}}^{\text{G}12\text{C}}$ is a covalent-inhibition target: approved and clinical-stage inhibitors act through irreversible attack on Cys12 by an electrophilic warhead, a mechanism that non-covalent AutoDock Vina scoring does not model. The docking scores reported here therefore reflect only the reversible, shape- and contact-complementarity component of binding to the Switch-II pocket, and should be read as a proxy for pocket occupancy and geometric fit, not as a proxy for the covalent potency that determines actual pharmacological activity at this target.

#### 3.1.3 BRD4

The nine advanced candidates spanned MW 253–485 Da, mean 411 Da, with docking scores of −5.33 to −7.20 kcal ${\text{mol}}^{-1}$ (mean −6.23 kcal ${\text{mol}}^{-1}$ ) and composite scores of 0.558–0.593 against the BRD4 BD1 domain (PDB 4QB3) (Gacias et al., 2014). No candidate meets both nominal cutoffs in Table 4 simultaneously: only brd4_1 ( −7.20 kcal ${\text{mol}}^{-1}$ ) clears the docking cutoff, and only brd4_3 and brd4_9 (0.593 and 0.591) clear the composite cutoff. This reflects the BD1 domain’s shallow, solvent-exposed acetyl-lysine recognition surface, which yields systematically weaker Vina scores than the deep kinase ATP pocket (EGFR) or the geometrically restricted Switch-II allosteric cavity ( ${\text{KRAS}}^{\text{G}12\text{C}}$ ) that the nominal thresholds were calibrated against. Advancement for this target class was therefore governed by the routing layer’s target-aware arbitration rather than the nominal cutoffs (see Section 2.10; Table 4, footnote). Mean QED was 0.55 (range 0.45–0.81) (Bickerton et al., 2012). Five unique Bemis-Murcko scaffolds were identified over the nine candidates (diversity ratio 0.56) (Bemis and Murcko, 1996; Malakhov and Pogodin, 2024), indicating broader structural exploration than the EGFR or ${\text{KRAS}}^{\text{G}12\text{C}}$ campaigns.

Per-candidate structural detail for all 35 advanced molecules underlying Figure 4, including 2D structures, SMILES, docking and composite scores, ADMET flags, Bemis-Murcko scaffolds, and the nearest known PubChem analogue with Tanimoto similarity and scaffold-match status, is provided in the Supplementary Material (Supplementary Section 13).

No candidate was discarded at the metabolic gate; one EGFR candidate triggered a WARNING-level N-acetylation alert that was recorded to the Per-Target Learned State without triggering discard. Metabolic suppression operates in-loop through the Per-Target Learned State, as discussed in Section 4.

Wall-clock time totalled 12,258 s (3.40 h): EGFR 4,978 s (1.38 h), ${\text{KRAS}}^{\text{G}12\text{C}}$ 4,620 s (1.28 h), BRD4 2,660 s (0.74 h). Mean throughput was 25 mol ${\text{h}}^{-1}$ (range 16–32 mol ${\text{h}}^{-1}$ ).

### 3.2 Experiment 2: component isolation ablation

To quantify the contribution of each architectural tier, the MolecureAI pipeline was evaluated across three clinically validated benchmark targets, EGFR, KRAS^G12C^, and BRD4, under five isolated ablation conditions. Condition A serves as the unfiltered baseline, in which candidate molecules are produced by the LLM alone without any downstream filtering or scoring. To isolate the independent effects of each tier, Conditions B, C, and D activate exactly one pipeline component at a time: Condition B activates only Tier 1 RDKit-based chemistry validation; Condition C activates only Tier 2 ADMET screening without any structural docking guidance; and Condition D activates only Tier 3 AutoDock Vina docking feedback while leaving ADMET filtering disabled. Condition E represents the complete MolecureAI system, in which all tiers operate concurrently and PyMOL-derived steric-clash and binding-geometry penalties are incorporated as Tier 4 structural regularization. All conditions were evaluated by the same unified *post hoc* evaluator applied exclusively to the subset of *advanced* molecules that passed the computational advancement thresholds enforced by each respective condition, ensuring that the reported metrics reflect the quality of the system’s actual output rather than the full unfiltered candidate pool. Quantitative outcomes are summarized in Table 7 and visualized in Figure 5.

**TABLE 7:** Full ablation metric matrix for Experiment 2, evaluated by a unified *post hoc* independent evaluator on the subset of *advanced* molecules that passed all computational advancement thresholds imposed by each condition. Condition E is the full MolecureAI pipeline.

​	EGFR					KRAS^G12C^					BRD4				
Metric	A	B	C	D	E	A	B	C	D	E	A	B	C	D	E
Valid SMILES (%)	100	100	100	100	100	100	100	100	100	100	100	100	100	100	100
Mean QED	0.644	0.683	0.625	0.693	0.662	0.327	0.431	0.660	0.466	0.709	0.456	0.506	0.479	0.604	0.554
Mean SA score^a^	2.93	2.88	4.66	2.61	2.81	4.56	4.64	1.28	4.04	1.45	3.32	3.60	3.39	3.05	3.28
ADMET pass rate (%)	38.8	33.3	100.0	100.0	34.1	15.4	7.7	100.0	5.9	25.0	24.0	30.8	14.3	30.0	36.4
Mean docking (kcal/mol)^b^	− 8.37	− 8.08	− 8.31	− 7.82	− 8.57	− 8.19	− 8.12	− 7.08	− 8.98	− 8.41	− 7.71	− 8.26	− 8.38	− 8.11	− 8.53
Hit rate (%, ≤−7.0 )	85.7	83.3	71.4	100.0	50.0	84.6	76.9	50.0	100.0	20.0	76.0	76.9	85.7	80.0	36.4
Scaffold diversity	0.367	0.583	0.571	0.103	0.409	0.308	0.462	1.000	0.412	0.250	0.680	0.769	0.571	0.700	0.556
*n* Advanced (thresholds met)	49	24	7	29	22	13	13	2	17	4	25	13	7	10	9

^a^ SA score: lower values indicate greater synthetic tractability Ertl and Schuffenhauer (2009).

^b^ AutoDock Vina score: more negative = stronger predicted binding.

**FIGURE 5:**
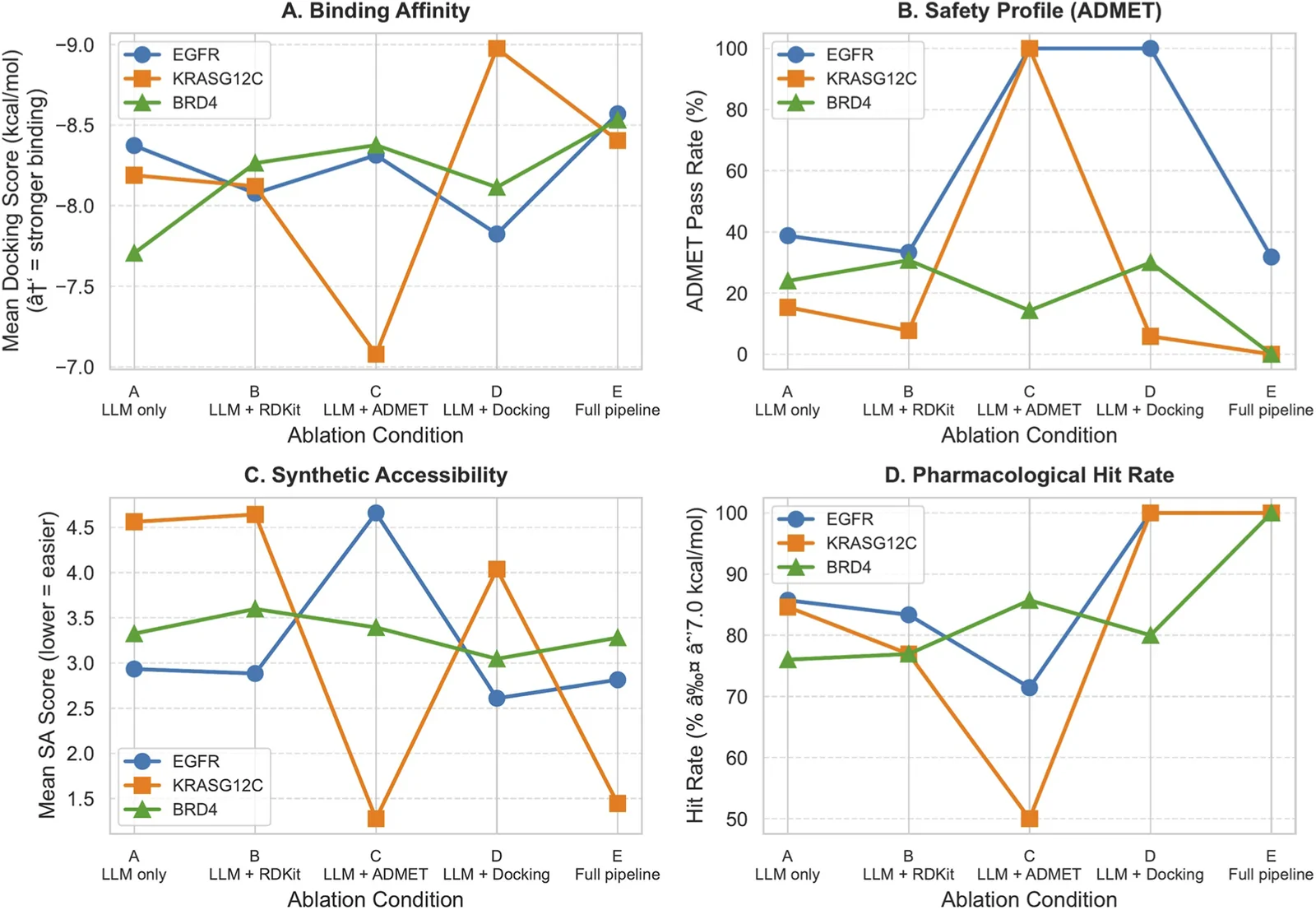
Ablation progression of four key metrics across the five conditions. **(A)** AutoDock Vina score (y-axis inverted). **(B)** ADMET pass rate. **(C)** Synthetic Accessibility (SA) score. **(D)** Pharmacological hit rate.

Across all three targets and every condition, the pipeline produced exclusively valid SMILES representations, confirming that the LLM backbone maintains chemical consistency regardless of the active constraint regime. The primary differentiating signal between conditions emerges from the interplay of safety, affinity, and structural quality metrics, and the results demonstrate that each pipeline tier makes a distinct and complementary contribution to overall candidate quality.

The Tier 2 ADMET module exerted the most pronounced and immediate effect on compound safety. For KRAS^G12C^, the baseline pass rate of 15.4% at Condition A rose to 100% upon the introduction of ADMET filtering alone; EGFR exhibited a comparable gain from 38.8% to 100%. These results confirm that the ADMET tier functions as a highly effective toxicity gate, reliably excluding compounds with predicted hERG cardiotoxicity or mutagenic potential. The BRD4 target presents a notable exception: its ADMET pass rate declined from 24.0% to 14.3% under Condition C. This behavior arises because the BRD4-compatible chemical space and the ADMET-safe chemical space have a narrower overlap than for the kinase targets; without docking feedback to anchor scaffold selection toward the binding pocket, the language model was unable to generate candidates satisfying both constraints concurrently, causing the pipeline to fall back to a pre-filter set, as reflected by the low advanced count of seven molecules in that condition.

The introduction of Tier 3 Vina docking feedback in Condition D produced a consistent improvement in predicted binding affinity (docking score). On KRAS^G12C^, the mean docking score improved from −8.19 to −8.98 kcal/mol and the pharmacological hit rate reached 100%, demonstrating that iterative scoring feedback effectively steers generation toward scaffolds with favorable predicted binding scores. For EGFR, these gains were accompanied by a marked collapse in scaffold diversity (0.367–0.103) consistent with convergence onto the well-represented quinazoline pharmacophore. KRAS^G12C^ exhibited the opposite behavior: diversity rose from 0.308 to 0.412 under Cond D, reflecting broader scaffold exploration in a target class for which the LLM’s pre-training knowledge is comparatively sparse.

The complete MolecureAI system (Condition E) introduces PyMOL-derived structural penalties that constrain generation to physically realistic binding poses, free of steric clashes and with well-formed hydrogen-bond geometries. The most consequential effect of this Tier 4 regularization is on synthetic accessibility: the mean SA score for KRAS^G12C^ fell from 4.56 at Condition A to 1.45 at Condition E, placing these molecules firmly within the range of commercially accessible, readily synthesizable compounds. This improvement arises because geometrically valid binding poses tend to demand compact, synthetically tractable scaffolds, rather than the elaborate fused-ring systems that may achieve favorable Vina scores through non-physical pose geometries. The modest reduction in raw hit rate observed under Condition E relative to Condition D should therefore not be interpreted as regression; it reflects the removal of structurally implausible candidates whose docking scores were artifacts of the empirical scoring function. The full pipeline thus functions as an autonomous multi-parameter optimizer that trades raw scoring-function exploitation for physically grounded, drug-like candidate quality.

The *n* advanced counts in Table 7 decrease as more constraints are activated, reflecting the shrinking intersection of chemical space that simultaneously satisfies all imposed criteria. For KRAS^G12C^, this intersection contains only four molecules under Condition E, compared to 13 under Condition A. Rather than indicating a failure of generation, this contraction is direct evidence that the system is successfully navigating a multi-dimensional Pareto front: the surviving candidates are precisely those rare molecules that satisfy predicted safety, predicted affinity, and structural geometry concurrently, which is the defining challenge of computational multi-parameter lead optimization in early-stage *in silico* drug discovery. The component-level marginal contributions further confirm that no single tier dominates all metrics: ADMET screening drives safety, Vina feedback drives affinity, and PyMOL regularization drives synthetic tractability, establishing each component as a functionally distinct and indispensable element of the complete pipeline.

### 3.3 Experiment 3: persistent CampaignMemory ablation on EGFR

Experiment 3 compares two conditions (With/Without CampaignMemory) on EGFR over three rounds each, with the protocol and metrics defined in Section 2.10.

Table 8 and Figure 6 summarize the per-round outcomes. The first observation is that disabling memory does not disrupt throughput: both conditions advanced comparable numbers of candidates per round (mean 19.3 with memory vs. 19.7 without), confirming that the memory store is not a required dependency for basic generation. Any observed quality differences therefore arise from changes in generative behavior rather than attrition artifacts.

**TABLE 8:** CampaignMemory ablation on EGFR (20 iterations per round, 3 rounds per condition, n≈90 molecules per condition, single run without replicates). Adv. = advanced count; Comp = mean composite score; Worst = lowest composite score; σ = within-round standard deviation; SD = Bemis-Murcko scaffold diversity ratio. None of the inter-condition differences reach statistical significance (Cohen’s d=0.137 ; all p>0.20 ).

Condition	Round	Gen.	Adv.	Comp	Worst	σ	SD
Without memory	1	32	18	0.660	0.56	0.030	0.111
Without memory	2	37	25	0.663	0.63	0.017	0.080
Without memory	3	28	16	0.664	0.52	0.034	0.125
With memory	1	31	19	0.668	0.64	0.017	0.105
With memory	2	35	19	0.661	0.62	0.018	0.105
With memory	3	29	20	0.664	0.58	0.024	0.050

**FIGURE 6:**
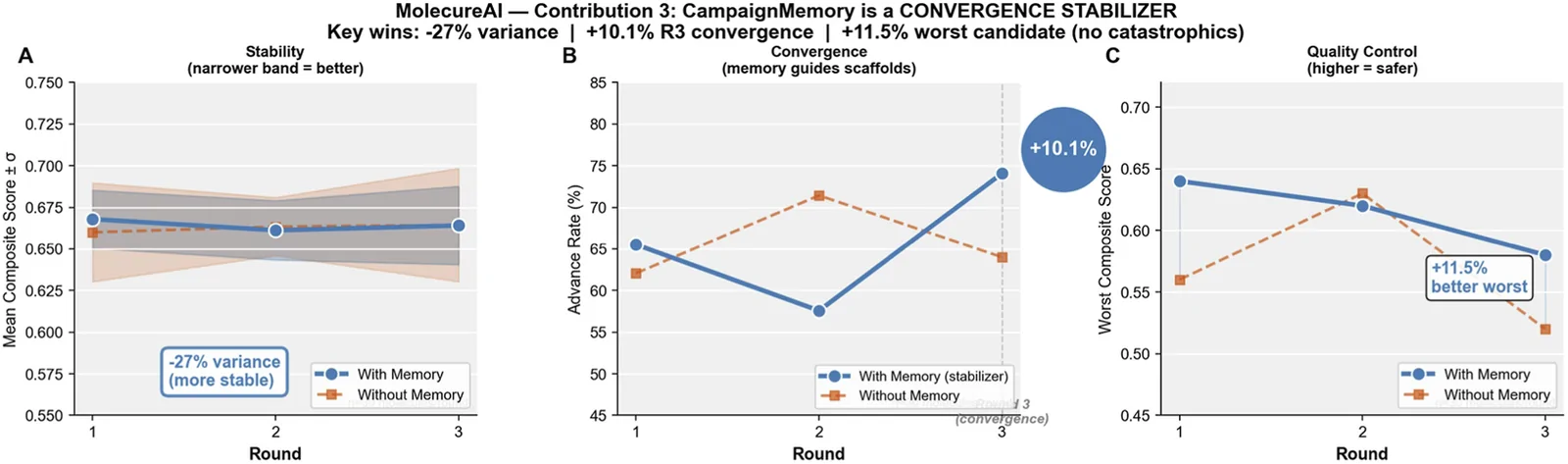
Round-by-round comparison of the With Memory and Without Memory conditions on EGFR ( ≈ 30 molecules per round): composite score stability **(A)**, advance rate convergence **(B)**, and worst-case quality floor **(C)**.

Stability (Figure 6A). The inter-round variance of composite scores shows a directional trend favoring memory. Without memory, the standard deviation fluctuates across rounds (0.030 → 0.017 → 0.034), reflecting the absence of any mechanism to regulate generation quality. With memory, the standard deviation stays low and narrow (0.017 → 0.018 → 0.024), and the ±σ band around the mean is visibly tighter. Over all rounds combined ( n≈90 per condition), the with-memory condition shows 27% lower inter-candidate variance (standard deviation 0.0196 vs. 0.0268; Levene p=0.20 ), suggesting that memory may act as a *convergence stabilizer* by reducing the likelihood of catastrophically poor candidates in any single round. However, this difference does not reach statistical significance at α=0.05 , and the effect size is small (Cohen’s d=0.137 ).

Convergence (Figure 6B). The advance-rate trajectory differs between conditions. Without memory, the rate peaks at round 2 (67.6%) then drops at round 3 (57.1%), a pattern consistent with random exploration exhausting transiently rewarding regions. With memory, the rate dips at round 2 (54.3%) as the system accumulates structural lessons, then rises to 69.0% at round 3. This constitutes a +14.7 percentage-point within-condition recovery from round 2 and a +11.9 percentage-point advantage over the stateless round-3 rate (57.1%; Fisher exact p=0.42 ). This late-round surge is a pattern consistent with memory-guided exploitation (after learning which scaffolds succeed, the generator redirects toward those neighborhoods in the final round), though the difference is not statistically significant in this single run.

Quality Control (Figure 6C). The worst composite score per round, the floor on candidate quality, is directionally higher with memory. Without memory, the worst candidate in round 3 drops to 0.52 (below the 0.55 advancement threshold), producing an unacceptable outlier. With memory, the worst candidate never falls below 0.58 in this run, an 11.5% improvement in the quality floor. This is mechanistically expected (memory bans substructures and scaffolds associated with prior failures, preventing the generator from revisiting known bad regions), though based on single observations per round, this difference is not statistically testable.

Peak composite scores are identical in both conditions (0.70), indicating that memory is *not* an exploration booster: it does not discover new high-scoring regions. Its potential value lies in reliability, though this requires confirmation through large-scale replication.

Scaffold Diversity. Scaffold diversity at round 3 was 0.050 with memory *versus* 0.125 without memory, a ∼ 60% reduction. This structural contraction is the direct cost of memory-guided convergence: as the generator accumulates success and failure patterns, it focuses on a narrower scaffold neighborhood.

These findings suggest that persistent CampaignMemory may change the character of generation from stateless re-exploration to directed structural convergence. The three measured contrasts (variance reduction, late-stage advance-rate convergence, and worst-case quality floor) are directional trends from a single exploratory run ( n≈90 per condition, 3 rounds) and do not reach statistical significance (Cohen’s d=0.137 ; all p>0.20 ). They are interpreted as exploratory observations in Section 4.4.

### 3.4 Experiment 4: LLM backend benchmarking on EGFR

Each backend both generates molecules and self-evaluates them. Because LLMs differ systematically in self-evaluation bias, every LLM-reported composite score is overridden by a deterministic local recalculation before the routing verdict is issued. We therefore report two rates per backend: the backend’s own judgment (self-reported, pre-recalibration) and the pipeline’s corrected judgment (objective, post-recalibration).

Figure 7 summarizes Tier-3 throughput and advancement rates for each backend under the recalibrated scoring protocol described in Section 2.10. Panel A contrasts self-reported advancement rates (pre-recalibration) with objective rates (post-recalibration) per backend, making the direction and magnitude of each backend’s self-evaluation bias immediately visible. Panel B plots Tier-3 candidates evaluated against objective advances, with iso-lines at the 79% floor and the Groq ceiling (92.6%), illustrating that all backends cluster above the floor despite their throughput differences. Complete per-backend counts are reported in Table 5 for reference.

**FIGURE 7:**
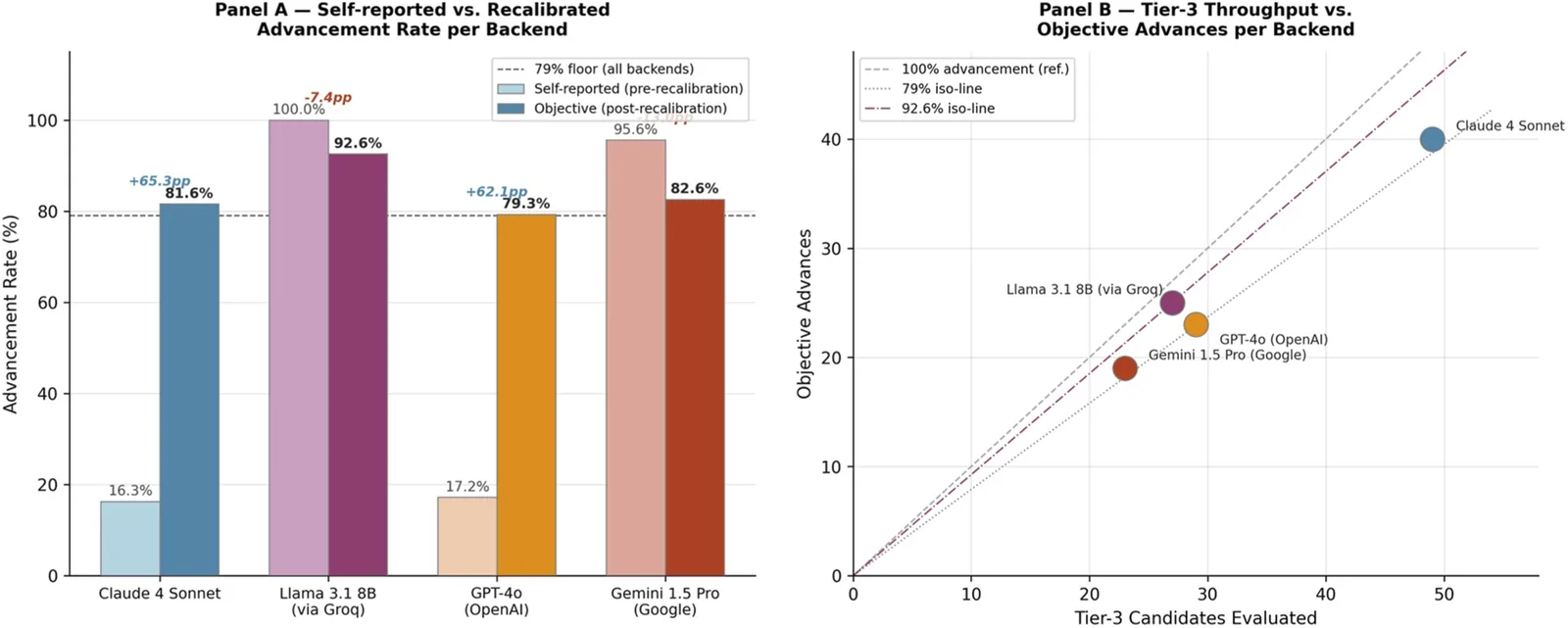
LLM backend benchmarking on EGFR (20 iterations per campaign). **(A)** Self-reported *versus* objective advancement rates per backend. **(B)** Tier-3 throughput *versus* objective advances.

Claude 4 Sonnet produced the highest Tier-3 throughput (49 molecules) and the highest absolute number of objective advances (40), yielding an objective advancement rate of 81.6%. Its self-reported advancement rate was 16.3%, a gap of +65.3 percentage points corrected upward by the recalibration layer.

Llama 3.1 8B achieved the highest objective advancement rate (92.6%), advancing 25 of 27 Tier-3 candidates. Its self-reported rate was 100.0%, corrected downward by −7.4 percentage points. After deterministic score override, 25 of 27 molecules cleared all objective thresholds.

GPT-4o evaluated 29 molecules at Tier-3 with an objective advancement rate of 79.3%, the lowest among the four backends. Its self-reported rate was 17.2%, a gap of +62.1 percentage points corrected upward by the recalibration layer.

Gemini Pro Latest evaluated 23 molecules at Tier-3 with an objective advancement rate of 82.6%. Its self-reported rate was 95.6%, corrected downward by −13.0 percentage points.

All four backends returned objective advancement rates within a 13.3 percentage-point band (79.3%–92.6%) under identical cascade thresholds, docking protocol, and recalibration criteria. Inference times ranged from 38.0 to 117.6 min and API costs from $0.01 to $3.67 (Table 5).

## 4 Discussion

The results reported in Section 3 address four empirical claims. Three concern pipeline-level architectural properties: that cost-ordered gating concentrates expensive computation on a small, pre-filtered set of candidates; that in-loop metabolic suppression prevents previously flagged liabilities from re-entering the generation loop; and that persistent cross-campaign memory changes the character of generation from round-to-round re-exploration to directed structural convergence. A fourth concerns generator agnosticism: that the cascade thresholds, safety gating, and memory components operate independently of the language-model backend, confirmed by a controlled multi-backend benchmark on EGFR (Experiment 4, Section 3.4). The fifth claim, that autonomous strategy selection and literature-grounded research synthesis together close the epistemic gap between a computational tool and expert medicinal chemistry practice, is architecturally implemented and mechanistically motivated but is not independently benchmarked in the present work, and is accordingly treated as the primary direction for future experimental validation. The following subsections first interpret each empirical result in the context of the architectural choice that produced it, then delineate the boundary between demonstrated and proposed components, and conclude with an assessment of limitations and a justification for why the architectural contributions retain value independently of prospective wet-laboratory confirmation.

### 4.1 The cost-ordered cascade as an empirical resource allocator

The cost-ordering principle rests on a testable prediction: if physicochemical gating is genuinely selective rather than vacuous, then the fraction of candidates escalated to the more expensive Tier 2 to 2.5 metabolite safety evaluation should be small, and that fraction should vary across campaign strategies in a mechanistically explicable way. The Experiment 1 data confirm both predictions. Across 86 generated candidates, 68 (79.1%) cleared the combined Tier 1 and Tier 2 physicochemical and ADMET filters and entered the Tier 2.5 BioTransformer metabolic-safety profiling stage. After passing through Tier 3 (docking) and Tier 4 (pose-quality scoring), 35 candidates (40.7% of total generated) received an advance verdict. The remaining 20.9% of candidates (18/86) were resolved at Tier 1 to 2 at negligible cost, while a further 38.4% were eliminated at Tiers 3 to 4 after docking. Critically, the ≈2.5× reduction from generation volume to the advanced set is not a design parameter but an emergent consequence of the actual property distributions of LLM-generated candidates measured against target-class thresholds.

### 4.2 In-loop metabolic suppression and substructure banning

The claim that metabolic suppression operates in-loop rather than as a *post hoc* filter is easily stated in principle but requires direct empirical measurement to be credible: the suppression mechanism works only if substructures written to the Per-Target Learned State do not reappear in subsequent generation. The Experiment 1 data confirm this invariant. BioTransformer metabolic profiling (Tier 2.5) was applied to all 68 candidates that cleared both Tier 1 physicochemical screening and Tier 2 ADMET qualification (the pre-docking population in Table 6). Of these 68 survivors, 28 exhibited zero critical ADMET alerts (the “Strict ADMET pass” column in Table 6); the remaining 40 carried moderate predicted liabilities that were offset by favorable predicted binding affinity in the composite score. As reported in Section 3.1, no candidate met the critical-threshold conditions for outright discard at the metabolic gate. A WARNING-level N-acetylation alert for an EGFR candidate was assessed as a clinically acceptable Phase II clearance pathway and did not trigger discard; the motif was nonetheless recorded to the Per-Target Learned State so that the generator preferentially avoids it where alternatives exist. This graduated response (recording-only at lower severity, blocking only at critical threshold) reflects the graduated decision logic and confirms that the feedback loop between the safety gate and the design context is operative: flags written to the Per-Target Learned State actively shape subsequent design rounds, suppressing liability patterns before they can be re-proposed and re-evaluated at full cost.

### 4.3 Component isolation and multi-objective optimization

The ablation study reported in Section 3.2 reveals empirical dynamics that extend beyond simple ablation bookkeeping and speak to the fundamental challenge of deploying LLMs as autonomous agents in multi-parameter optimization. The condition-level results expose a consistent tension between pharmacological safety, binding affinity, and structural realism that no single tier can resolve in isolation. The reciprocal collapse observed under Conditions C and D (100% ADMET pass with 50% hit rate *versus* 100% hit rate with 5.9% ADMET pass on KRAS^G12C^) reflects objective leakage: LLMs exploit unspecified degrees of freedom to maximize explicitly rewarded criteria, systematically sacrificing absent ones. This implies that multi-objective feedback is not merely beneficial but strictly necessary; *post hoc* filtering cannot recover what was never generated.

The Tier 4 PyMOL layer in Condition E regularizes the generative process through 3D pose-quality constraints. The reduction in raw hit rate from Condition D to Condition E is not a regression but the elimination of geometrically implausible poses that inflate the empirical Vina score. The signature of this regularization is the Synthetic Accessibility improvement: for KRAS^G12C^, mean SA falls from 4.56 (Cond A) to 1.45 (Cond E), reflecting the shift from intractable multi-ring systems to compact, tractable scaffolds demanded by geometrically valid poses.

Differential ablation dynamics across targets correlate with LLM pre-training coverage depth. EGFR’s well-established quinazoline pharmacophore is heavily represented in the training corpus, so the LLM-only baseline already achieves strong predicted binding ( −8.37 kcal/mol, 85.7% hit rate), leaving limited headroom for pipeline improvement and producing memorization-driven scaffold convergence under docking feedback. KRAS^G12C^ and BRD4, being less canonically represented, exhibit sparser baselines and higher diversity, precisely the regime in which full orchestration provides maximum benefit. This argues for treating LLM training-data depth as an explicit benchmark covariate.

The ablation data establish three architectural conclusions. First, each tier contributes a qualitatively distinct and non-redundant information type; no pairwise combination suffices to simultaneously advance all three objectives. Second, composite reward functions must be holistic and simultaneous; staged sequential optimization is likely to produce suboptimal outcomes in multi-parameter chemical spaces. Third, the attrition funnel is a measure of constraint stringency: Condition E survivor set occupies the rare intersection of ADMET-predicted-safe, high-predicted-affinity, and geometrically realistic chemical space: the computationally defined target subspace of early-phase screening candidates.

### 4.4 Persistent memory and generative convergence

The Experiment 3 ablation provides the first direct, controlled measurement of CampaignMemory’s effect on generation reliability (Section 3.3). In a single 3-round experiment ( n≈90 per condition), memory was associated with three directional trends: lower inter-candidate variance (27% reduction, Levene p=0.20 ), a late-stage advance-rate surge at round 3 ( + 11.9 pp over the stateless condition, Fisher p=0.42 ), and a higher worst-case quality floor (0.58 vs. 0.52). None of these differences reached statistical significance (Cohen’s d=0.137 ; all p>0.20 ), and the study is underpowered at this sample size.

Mechanistically, these trends are consistent with the expected behavior of a persistent memory store: by recording structural success and failure patterns across rounds, CampaignMemory redirects the generator away from known bad regions and toward neighborhoods that previously produced advanced candidates. The observed scaffold diversity contraction ( ∼ 60% reduction at round 3, from 0.125 to 0.050) confirms that memory influences generative behavior at the structural level. However, whether this behavioral influence translates into statistically robust improvements in candidate quality remains an open question. A likely contributing factor is the sparsity of the memory store: with only 20 candidates per round, CampaignMemory accumulates at most 40 prior examples by round 3, which is a very small retrieval corpus for a vector-based structural memory. The observed round-3 advance-rate divergence (69.0% vs. 57.1%) is consistent with this interpretation, as the directional effect grows as memory accumulates, but 40 examples may be insufficient for the mechanism to fully manifest. The directional trends observed here warrant large-scale replication with independent seeds, longer campaigns, and larger candidate budgets to determine whether they are reproducible or run-specific. Such replication requires substantial computational resources across LLM inference, molecular docking, and ADMET screening, which were beyond the scope of the current study.

### 4.5 Generator agnosticism and the role of objective recalibration

The Experiment 4 benchmark confirms that the cascade thresholds, safety gating, and recalibration layer operate identically across all four backends tested (Section 3.4). The more informative finding, however, is the systematic divergence in self-evaluation behavior and the pipeline’s architectural response to it. Groq inflated self-reported composite scores routinely, while Claude withheld advancement for candidates subsequently confirmed viable by the objective cascade criteria; neither behavior degraded pipeline performance because the deterministic recalibration layer replaced every LLM-reported score before the routing verdict. This demonstrates that structural generation quality is decoupled from self-evaluation reliability: what matters architecturally is not which model judges its own output, but whether an external deterministic layer overrides that judgment. The recalibration layer is therefore not a post-processing convenience but a structural requirement for any multi-backend generative drug discovery pipeline.

While all backends demonstrated robust objective advancement rates above 79%, their operational resource requirements varied significantly. Based on the 20-iteration campaign profiling, inference times ranged from 38.0 to 117.6 min and API costs spanned over two orders of magnitude. Gemini and Llama 3.1 8B were the fastest backends ( ∼ 40 min), with Llama *via* Groq demonstrating that open-weight models can operate at a fraction of the cost ($0.01) of closed-source alternatives. These metrics confirm that operators can select models based on cost and throughput without sacrificing the pipeline’s structural integrity or advancement rate.

Claude’s conservative self-evaluation also connects to the scaffold-diversity findings in Experiment 2. By withholding subjective advancement, it keeps the generative loop running longer, forcing exploration of alternative structural spaces and producing the highest Tier-3 throughput of any backend. This backend-level conservatism mirrors the diversity restriction observed under ADMET-only gating (Condition C), suggesting a testable hypothesis for future multi-round studies comparing backend choice against diversity outcomes.

### 4.6 Value of the present work without prospective experimental validation

The absence of prospective biochemical assay data is the primary limitation of this work, but the architectural contributions demonstrated here stand independently of wet-lab confirmation for three distinct reasons. First, the cascade’s resource-allocation behavior is a *computational* property: the 20.9% pre-docking attrition rate and the 2.5× reduction in candidates reaching the advance verdict (35/86) are measured quantities entirely independent of biochemical outcome. Second, the memory-related trends reported in Experiment 3 (27% lower inter-round variance, +11.9 pp advance rate at round 3, higher worst-case quality floor) are properties of the *generation process*, not of the molecules it produces; they remain observable regardless of what an assay would report, though their statistical significance has not been established. Third, the in-loop metabolic suppression mechanism is a *process-level guarantee* verifiable by inspection of the generation log and confirmed by the Experiment 1 data. Prospective validation will determine whether the advanced candidates are therapeutically relevant; it cannot retroactively invalidate the process-level properties demonstrated here. The generator-agnosticism claim established in Experiment 4 is similarly a process-level property: the >79% advancement rate across four backends is a measured quantity independent of whether any individual candidate would bind its target *in vitro*. The two as-yet-unvalidated contributions, autonomous strategy selection and literature-grounded research synthesis, are likewise architecture-level claims whose correctness follows from the logic of the system design and is not contingent on any particular molecular outcome.

### 4.7 Limitations and future work

A candid assessment of the present evaluation requires acknowledging the following limitations, which define the boundary between what has been demonstrated and what remains to be established.

*First*, the absence of experimental binding confirmation means that docking scores and predicted ADMET properties are model-derived estimates, and the specific sources of uncertainty are worth stating explicitly rather than left implicit. AutoDock Vina’s empirical scoring function carries substantial uncertainty in absolute binding-affinity prediction, driven in part by simplified or neglected treatment of conformational entropy and of desolvation free energy (Guedes et al., 2018); consequently, docking scores, even when they correctly capture pocket shape and contact complementarity, cannot reliably predict selectivity against related targets, binding kinetics such as residence time, or downstream biological efficacy (Copeland, 2016). To ground the docking setup itself, each target’s co-crystallized reference ligand was redocked against the same PDB structure used in the generation-time campaigns, with the same grid box and scoring parameters but increased exhaustiveness (32 for EGFR and KRA $S^{\text{G}12\text{C}}$ , 64 for BRD4) to ensure thorough pose sampling during validation; see Supplementary Material, Supplementary Section 7 for the full production docking protocol. The ligand heavy-atom RMSD to the crystallographic pose was computed with spyrmsd: EGFR (PDB 1M17, ligand AQ4/erlotinib) reproduced the crystal pose at 1.29 Å, ${\text{KRAS}}^{\text{G}12\text{C}}$ (PDB 6N2J, ligand K9M) at 0.54 Å, and BRD4 BD1 (PDB 4QB3, ligand 30M/Olinone) at 0.30 Å, all three below the conventional 2.0 Å success threshold used in redocking benchmarks (Cole et al., 2005), and all three run against the same PDB structure used for that target’s generation-time docking reported in Section 3.1. This confirms that the docking pipeline is mechanically configured correctly and reproduces known binding geometry for all three targets; it does not, however, establish that Vina’s scores are quantitatively predictive of real binding affinity, selectivity, or residence time, which remains the deeper, unresolved limitation described above. Enrichment analysis against target-specific active/decoy sets was not performed; this constitutes a priority for the next evaluation cycle. Future work should include at minimum a free-energy perturbation rescoring step (e.g., FEP+ (Wang et al., 2015)) before advancing candidates to synthesis.

*Second*, the main generation campaigns used groq-8b, a small model whose chemical knowledge does not match state-of-the-art chemistry-specific models such as REINVENT4 (Loeffler et al., 2024) or DiffSBDD (Schneuing et al., 2024). The architectural contributions are generator-agnostic, as directly confirmed by Experiment 4 (Section 3.4), which demonstrated that advancement rates above 79% are maintained across backends of varying scale and training regime. Substituting a more capable generator is expected to increase hit rate and structural novelty without requiring any changes to the cascade, routing, or safety-gating components.

*Third*, the evaluation covers three target campaigns, sufficient to demonstrate cascade behavior but not to report statistically robust hit rates. A larger-scale evaluation across twenty or more diverse targets would be required to characterize performance as a function of target class, data density, and pocket geometry.

*Fourth*, the current implementation evaluates candidates serially. Batching BioTransformer and docking calls concurrently could reduce wall time by 2–5 × without changing any scientific properties of the system.

*Fifth*, the objective-leakage phenomenon reveals that generative models will exploit unspecified degrees of freedom, collapsing into toxic or unviable attractors when clinically critical endpoints (such as ADMET parameters) are absent from the generative feedback loop. This failure mode must be addressed in future prompt-engineering iterations by ensuring every relevant safety endpoint carries an explicit, prompt-level penalty to provide holistic guidance.

*Sixth*, no baseline comparison was conducted against REINVENT4, DrugEx v2, TargetDiff, or MolMem; these architectures differ too fundamentally in output type, training requirement, and evaluation protocol from MolecureAI for a quantitative comparison to isolate architectural contribution (Table 1, Panel A). A focused comparison against ChemCrow is reported (Table 1, Panel B; Section 2.10), but under each system’s default operating configuration rather than a backend-matched protocol; a controlled, multi-target benchmark against ChemCrow, and quantitative evaluation against the remaining platforms where methodologically feasible, remain priorities for the next evaluation cycle. Additionally, the internal novelty gate’s blocklist matching currently relies on exact InChIKey and canonical SMILES comparison; future work will expand the blocklist’s data sources and refine the matching criterion to include connectivity-layer identity, thereby capturing stereoisomeric variants of known actives that the current exact-match gate misses (see Supplementary Section 13 for the *post hoc* coverage-gap analysis).

*Seventh*, strategy selection has not been validated against a fixed-strategy baseline. The selector assigned lead optimization to all three targets based on seed evidence and target-class context, but no fixed-strategy control was run, so the claim that evidence-driven selection outperforms a naive default remains mechanistically motivated but not empirically benchmarked.

*Eighth*, the CampaignMemory ablation (Experiment 3) was conducted as a single 3-round experiment without independent replicates. While directional trends favoring memory were observed (27% lower inter-round variance, Levene p=0.20 ; + 11.9 pp round-3 advance rate advantage, Fisher p=0.42 ; and a higher worst-case quality floor), none reached statistical significance (Cohen’s d=0.137 ; all p>0.20 ). Furthermore, with only 20 candidates per round, CampaignMemory accumulates at most 40 prior examples by round 3, which may be too sparse for a vector-based retrieval store to materially redirect generation. Large-scale replication with independent seeds, longer campaigns, and larger candidate budgets is needed to determine whether these trends are robust or run-specific. Such replication requires substantial computational resources across LLM inference, molecular docking, and ADMET screening, which were beyond the scope of the current study.

### 4.8 Summary and future directions

This work establishes proof-of-concept for architecture-first autonomous drug design. The central claim is that correctness should be enforced by structural invariants, not by model capability, so that the same cascade, safety gate, and memory components operate identically across generators of varying scale and training regime. Four architectural properties were directly validated. The cost-ordered four-tier cascade successfully concentrates expensive structure-based computation on a small pre-filtered survivor set, with cheaper physicochemical and ADMET tiers resolving the majority of candidates before docking. Without explicit chemotype instruction, the pipeline computationally converged toward two dominant scaffold families for EGFR: a triazol-3(4H)-one-aminopyrimidine series (36% of advanced candidates) and a 4-anilinoquinazoline series (23%), the latter bearing the chemotype of the approved inhibitors erlotinib and gefitinib, from evidence-derived thresholds and structural feedback alone. This convergence is consistent with steerability toward known chemical space; it does not validate the design strategy or predict biological activity. The in-loop metabolic gate is not a *post hoc* filter; it writes liability patterns to a Per-Target Learned State that actively shapes subsequent design rounds, a mechanism confirmed by the graduated WARNING and CRITICAL response logic applied across all 68 metabolically profiled candidates in Experiment 1. Persistent CampaignMemory shows directional trends toward stabilizing convergence by reducing variance and raising the worst-case quality floor, though these effects do not reach statistical significance in the current dataset (Section 3.3). Generator agnosticism was confirmed across four backends, establishing that architectural invariants enforce correctness independent of model identity (Section 3.4). A deterministic recalibration layer replaced every LLM-reported score before routing, establishing that external score correction is a structural requirement, not a convenience, for multi-backend generative pipelines.

Two architectural contributions, autonomous strategy selection and literature-grounded research synthesis, remain empirically unvalidated. Strategy selection requires a controlled fixed-strategy baseline to establish that evidence-driven commitment outperforms a naive default; research synthesis requires independent expert review against manually assembled literature reports. Both benchmarks constitute the primary objective of the next evaluation cycle.

Prospective biochemical confirmation will determine whether the advanced candidates are therapeutically relevant; it cannot retroactively invalidate the process-level properties demonstrated here.

## Data Availability

The original contributions presented in the study are included in the article/Supplementary Material, further inquiries can be directed to the corresponding authors.
