# Supplementary material for: MolecureAI: an agentic AI platform for autonomous drug discovery
Source: Front Pharmacol. 2026 Sep 29;17:1917774. doi: 10.3389/fphar.2026.1917774 (PMC13658513; doi:10.3389/fphar.2026.1917774)
Supplement: Supplementary file 1 [file DataSheet1.pdf]

Supplementary Material

1 COMPUTATIONAL ENVIRONMENT

All experiments were executed on a single consumer-grade CPU workstation with no GPU acceleration. The operating system was Ubuntu 22.04 LTS. Python 3.11 was used as the runtime environment.

2 QUERY DECOMPOSITION RULES AND SOURCE-TO-SECTION MAPPINGS

A research campaign decomposes the user query into five subquestions, each dispatched through the MCP tool layer to a canonical source:

1. **Target biology** - UniProt, Ensembl, and Open Targets for protein identity, genomic context, and target-disease associations.
2. **Known actives with annotated liabilities** - ChEMBL for bioactivity records (IC_{50}, K_i, K_d).
3. **Clinical precedent** - ClinicalTrials.gov for trial outcomes, status, and phase progression.
4. **IP landscape** - Patent feed queries for freedom-to-operate assessment.
5. **Synthesis narrative** - Convergent findings and open questions assembled by the reasoning layer.

Each subquestion is routed to its source by keyword matching on the query intent. The report sections are: (1) Target Overview, (2) Known Actives and Annotated Liabilities, (3) Clinical Precedent, (4) Intellectual Property Landscape, (5) Synthesis Narrative. Every factual claim carries a traceable data-source citation.

3 SOFTWARE AND TOOL VERSIONS

Table S1. Core software dependencies and versions used in all experiments.

Component	Version	Purpose
RDKit	$\geq$2023.9.1	Cheminformatics (SMILES validation, QED, scaffolds)
AutoDock Vina	$\geq$1.2.5	Structure-based docking
Meeko	$\geq$0.5.0	Ligand preparation (SMILES $\rightarrow$ PDBQT)
BioTransformer	3.0	Metabolic profiling and metabolite prediction
ADMET-AI	v1	ADMET property prediction (cardiac, mutagenic, BBB, solubility)
PyMOL	$\geq$2.5.0	Structural validation and pose quality analysis
Python	$\geq$3.11	Runtime environment

4 LLM BACKEND CONFIGURATIONS

All generation campaigns in Contributions 1-3 used `groq-8b` (Groq-hosted Llama 3.1 8B Instant) as the unified reasoning, generation, and routing engine. This model was selected for its sub-second token latency on the Groq inference hardware, which allowed tight generation-evaluation-routing loops without GPU infrastructure on the experimental host.

The LLM backbone benchmark (Contribution 4) evaluated four backends under identical architectural conditions, selected to span the commercial and open-weight capability spectrum: a small open-weight model (`groq-8b`), a mid-size proprietary model (`gpt-4o`), a large proprietary model (`claude-sonnet`), and a frontier multimodal model from a separate provider (`gemini-1.5-pro`). All four backends received identical system and user prompts; cascade thresholds, docking parameters, and evaluation metrics were held constant.

Table S2. LLM backends evaluated in the backbone benchmark (Contribution 4). Model identifiers reflect the API strings used at inference time.

Label	Provider	Model identifier	API endpoint
`groq-8b`	Groq	llama-3.1-8b-instant	Groq Inference API
`claude-sonnet`	Anthropic	claude-sonnet	Anthropic Messages API
`gpt-4o`	OpenAI	gpt-4o	OpenAI Chat Completions API
`gemini-1.5-pro`	Google	gemini-1.5-pro	Google Generative Language API

5 MOLECULAR GENERATION CAMPAIGN PARAMETERS

5.1 Contribution 1: Cascade Validation

One campaign was executed per target (EGFR, $KRAS^{G12C}$, BRD4), consisting of twenty generation iterations. For each target, eight seed molecules were retrieved from ChEMBL with heterogeneous quality profiles. All three targets were run under lead-optimization constraints (Ro5-compliant seeds, narrow target-class neighborhoods), as the autonomous strategy selector committed to optimization mode for each campaign based on the seed evidence. Results across all iterations were deduplicated before reporting aggregate statistics.

5.2 Contribution 2: Component Isolation Ablation

To quantify the contribution of each architectural tier, the pipeline was evaluated across all three targets under five progressively structured conditions. Each condition introduced one additional component in isolation:

- **Cond A (LLM only):** Unfiltered baseline; the LLM receives only target biological context (identity, disease associations, and known inhibitor landscape retrieved from the evidence layer) with no explicit physicochemical constraints and no downstream filtering or scoring.
- **Cond B (+Tier 1 RDKit):** Tier 1 physicochemical validation active (drug-likeness, synthetic accessibility, pan-assay interference).
- **Cond C (+Tier 2 ADMET):** Tier 2 ADMET screening active without any structural docking guidance.
- **Cond D (+Tier 3 Docking):** Tier 3 AutoDock Vina docking feedback active while ADMET filtering remained disabled.
- **Cond E (Full pipeline):** Complete system with all tiers active, including Tier 4 PyMOL-derived steric-clash and binding-geometry penalties.

5.3 Contribution 3: CampaignMemory Ablation

A controlled ablation on EGFR compared two conditions over three rounds each, with 20 iterations per round (approximately 30 candidates per round):

- **Without Memory:** The Qdrant-backed Evaluation Memory store was disabled by pointing the client to an invalid host.
- **With Memory:** The full Qdrant-backed store was active, persisting embeddings, success/failure patterns, and banned substructures across rounds.

All other parameters (model, cascade thresholds, docking protocol, BioTransformer, evaluation metrics) were held constant.

5.4 LLM Backbone Benchmark

For each of the four LLM backends (Table S2), 20 iterations were run against EGFR (PDB 1M17) while holding all cascade thresholds, docking protocol, BioTransformer configuration, and evaluation metrics identical to the main experiments.

6 TARGET PDB STRUCTURES AND PREPARATION

Protein structures were retrieved from the Protein Data Bank and prepared as follows:

- **EGFR:** PDB 1M17 (EGFR wild-type + erlotinib, 2.00 Å); grid centered on the ATP-binding cleft.
- **KRAS^{G12C}:** PDB 6N2J (allosteric pocket, 1.80 Å); grid centered on the covalent inhibitor binding site.
- **BRD4:** PDB 4QB3 (bromodomain 1, 0.94 Å); grid centered on the acetyl-lysine recognition pocket.

Protein structures were prepared with PDBFixer/OpenMM (addition of hydrogen atoms at pH 7.4, removal of heterogens). Ligand protonation at pH 7.4 was handled by Meeko during SMILES-to-PDBQT conversion. Grid boxes were sized to 20-30 Å per edge, centered on the co-crystallized ligand center of mass. Missing side chains were not rebuilt; the pipeline used the deposited coordinates as-is.

7 DOCKING PROTOCOL

Ligand preparation was performed with Meeko, converting each candidate SMILES to PDBQT format with default protonation at pH 7.4. AutoDock Vina was invoked with the parameters listed in Table S3; only the top-ranked pose was retained per candidate.

Table S3. AutoDock Vina docking parameters applied uniformly across all targets and contributions.

Parameter	Value	Override variable	Description
Grid box size	20-30 Å	N/A	Derived from co-crystallized ligand extent
Grid center	Ligand COM	N/A	Center of mass of reference ligand
Exhaustiveness	4	`VINA_EXHAUSTIVENESS`	Search depth (Monte Carlo steps)
Number of poses	3	`VINA_N_POSES`	Top 3 poses retained; best scored

8 ADMET AND SAFETY GATING

ADMET property estimation was performed by ADMET-AI across cardiac, mutagenic, blood-brain-barrier, solubility, and metabolic-inhibition endpoints. The Tier 2 gate applied the following signal classification:

Table S4. Tier 2 ADMET signal thresholds for hERG and AMES.

Endpoint	GOOD	WARNING	CRITICAL
hERG (probability)	≤0.50	>0.50 - <0.90	≥0.90
AMES (probability)	≤0.50	>0.50 - <0.80	≥0.80

9 METABOLIC PROFILING

BioTransformer 3.0 was invoked for all candidates that passed Tier 2 ADMET gating. The tool predicted phase-I and phase-II metabolites, and any metabolite bearing a critical safety signal (reactive or mutagenic) triggered substructure banning in the Per-Target Learned State store.

10 COMPOSITE SCORING WEIGHTS

Table S5. Default composite score weights. Weights renormalize to 1.0 when a component is unavailable.

Component	Weight	Rationale
ADMET safety	0.23	Predicted toxicity dominates clinical attrition
Regulatory liability	0.07	Hepatotoxicity and metabolic inhibition vs. FDA priors (Sec. 2.7)
Structural relevance	0.19	Pose-level contacts and complementarity
Drug-likeness (QED)	0.20	Overall developability score
Docking affinity	0.17	Vina binding energy, target-class normalized
Solubility	0.09	Aqueous solubility prediction
Synthetic accessibility (SA)	0.05	Ease of synthesis estimate

11 OBJECTIVE SCORING RECALIBRATION

Preliminary log analysis revealed systematic self-reporting biases across LLM backends. To neutralize these biases, all LLM-reported scores were overridden by a deterministic local recalculation that applies the fixed weight vector in Table S5 to objective, externally computed property values.

A candidate was marked *Advanced* if and only if it jointly satisfied all three of the following criteria:

$$\text{Composite score} > 0.59 \tag{S1}$$

$$\Delta G_{\text{Vina}} \leq -7.0 \text{ kcal/mol} \tag{S2}$$

$$N_{\text{ADMET,critical}} = 0 \tag{S3}$$

where $N_{\text{ADMET,critical}}$ denotes the number of critical-tier ADMET alerts (hERG ≥ 0.90 or AMES ≥ 0.80). All three conditions must hold simultaneously, subject to the target-class normalization described below.

The composite and docking thresholds above (Eq. S1-S2) are nominal defaults calibrated on deep enzymatic pockets (kinase ATP sites, Switch-II allosteric cavities). For targets with shallow, solvent-exposed binding surfaces, such as the BRD4 BD1 acetyl-lysine recognition site, Vina scores are systematically weaker at equivalent binding quality, and the routing layer's target-aware arbitration admits candidates below these nominal cutoffs. This is why the nine BRD4 candidates in Table S12-S13 report docking scores of -5.33 to -7.20 kcal/mol and composite scores of 0.558 to 0.593, below the nominal -7.0 kcal/mol

docking cutoff for all but one candidate (`brd4_1`) and below the nominal 0.59 composite cutoff for all but two candidates (`brd4_3`, `brd4_9`); no candidate clears both cutoffs simultaneously. EGFR and $KRAS^{G12C}$ candidates (deep pockets) uniformly satisfy both nominal thresholds.

12 LLM GENERATION PROMPT ARCHITECTURE AND SAMPLING SETTINGS

12.1 Sampling Parameters

All LLM backends share a sampling temperature of 0.2; top-p, max tokens, and timeout differ by backend (Table S6).

Table S6. LLM sampling parameters applied across all backends and contributions. All backends use temperature 0.2.

Parameter	Value	Notes
Temperature	0.2	All backends; low-temperature for deterministic output
Top-p	0.9	Groq, GPT-4o, Claude; Gemini uses SDK default
Max tokens	2000 (groq-8b)	Overridden from 1024 for campaign generation calls
	1200 (groq-70b)	Configurable via environment variable
	32000 (GPT-4o, Claude)	
	8192 (Gemini)	Configurable via environment variable
Request timeout	60 s (groq-8b)	
	120 s (groq-70b)	
	300 s (Claude direct)	
Max retries	10	Groq backends; automatic retry on rate-limit errors

12.2 Prompt Assembly Architecture

Each generation call assembles a structured prompt from five components drawn from the campaign state, as described in Section 2.3 of the main text. It begins with the strategy context (active mode, target class, and PDB identifier), followed by the design anchor (seed molecules with ChEMBL identifiers and IC_{50} values together with Murcko scaffold hints). Next, the prompt incorporates retrieved memory (Qdrant-backed semantic retrieval of past evaluations, banned substructures, and per-target learned state), then avoidance constraints (previously generated SMILES and hard-filter rejection reasons from the prior round), and finally property targets (mode-specific MW/LogP/TPSA windows, ADMET thresholds, and generation contract constraints from the Layer 3 planner).

The prompt is structured into the following sections, assembled in fixed order:

1. **Context line**: target gene, target class, PDB identifier.
2. **Hard property limits**: target-class-specific rules (e.g. MW 300-550 Da, LogP 1-5 for lead optimization; MW 120-280 Da, LogP <3 for FBDD) with explicit discard thresholds.
3. **Seed molecules**: up to 5 ChEMBL actives (MW $\leq$ 450 Da) formatted as `SMILES | ChEMBL_ID | IC50 nM`, serving as structural starting points.
4. **Conditional sections** (injected when triggered by campaign state; see Section 12.3 below).
5. **Campaign state**: current iteration number, up to 12 previously generated SMILES to avoid, and the memory block (when enabled).
6. **Task specification**: exact number of SMILES to generate, mode-dependent task line, similarity requirement, and diversity constraints.

7. **SMILES validity rules**: explicit instructions for ring closure, parenthesis/bracket balance, chemical reasonableness, and duplicate avoidance.
8. **JSON output format**: structured response schema requiring a strategy list and a candidates array, where each candidate includes SMILES, seed derivation, modification applied, and expected improvement.

12.3 Conditional Prompt Sections

Several prompt sections are injected conditionally based on campaign state and mode:

- **Scaffold hint** (lead-optimization mode, when seeds are available): Murcko scaffolds extracted from ChEMBL seed molecules, instructing the LLM to preserve validated core ring systems and modify substituents only.
- **FBDD fragment design brief** (fragment-based mode): Rule of 3 property envelope (MW 120-280 Da, LogP < 3, HBD/HBA ≤ 3) and a list of privileged fragment scaffolds (aminopyrimidine, benzimidazole, indole, 7-azaindole, etc.).
- **Failure feedback** (iteration > 1, when hard-filter rejections occurred): Up to 6 rejection reasons from the previous round, with mandatory avoidance instructions. When $>$50% of failures are hERG-related, an additional cardiotoxicity-awareness section with 8 medicinal-chemistry rules is injected.
- **Fix section** (when repair candidates exist): Up to 3 molecules flagged for structural correction, with specific failure reasons, to be modified before generating new candidates.
- **Memory block** (when memory is enabled): Qdrant-retrieved semantic context (top-5 similar past evaluations by ChemBERTa similarity, per-target learned state, banned substructures), capped at 2000 characters.
- **Generation contract constraints** (Layer 3 output): MW/LogP ranges, scaffold exclusions, Tanimoto diversity minimums, and fragment-growing anchor SMARTS, injected as explicit constraints from the planner's per-round strategy.

12.4 Illustrative Generation Prompt

The following shows a representative prompt structure for a lead-optimization round, populated with illustrative (non-proprietary) values. Curly-brace fields are filled at runtime from the campaign state. The verbatim production prompt templates are withheld as proprietary intellectual property (see Data Availability Statement); this structural specification is provided to enable assessment and largely reproduce the generation methodology.

```
# Molecular Design Generation Agent
{scaffold_hint}{failure_feedback}{mutation_example}

## Context
Target: EGFR (kinase) | PDB: 1M17

## Hard Property Limits (violating these causes immediate discard)
  - MW: 300-550 Da (HARD LIMIT: discard if > 560 or < 250)
  - LogP: 1-5 (HARD LIMIT: discard if > 6)
  - HBD < 5, HBA < 10
  - TPSA < 140 Angstrom^2
```

```
## Seed Molecules (real actives - structural starting points)
  COc1cc2ncnc(Nc3ccc(F)c(Cl)c3)c2cc1OCCCN1CCOCC1 | CHEMBL3881 | 2.0 nM
  (Format: SMILES | ChEMBL ID | IC50 nM)

## Campaign State
Iteration: 3 | Past SMILES to AVOID: [SMILES_1, SMILES_2, ...]

## Task
Generate exactly 10 novel SMILES, one per candidate.
Each candidate MUST be a close structural analogue of a seed
molecule (Tanimoto > 0.4).

## Strict Generation Rules (CRITICAL)
1. RDKit Validity: Every SMILES must be chemically valid.
2. Parenthesis Balance: count '(' and ')' - they MUST be equal.
3. Bracket Balance: count '[' and ']' - they MUST be equal.
4. Synthesisability: avoid multi-fused rings (SA-Score < 6.0).
5. No Duplicates: do not repeat any SMILES from past rounds.
6. Chemical Reasonableness: avoid unstable functional groups.

## Output: JSON only, no markdown
{
  "strategy": ["swapped methoxy for ethoxy on seed 1"],
  "candidates": [
    {
      "smiles": "...",
      "derived_from": "CHEMBL3881",
      "modification_applied": "replaced -OCH3 with -OC2H5",
      "expected_improvement": "improves metabolic stability"
    }
  ]
}
```

12.5 SMILES Validation and Repair Rules

1. **Canonicalization**: Every generated SMILES string is parsed and re-emitted in canonical form. Strings that fail to parse are discarded immediately and never enter the evaluation cascade.
2. **Deduplication**: Canonical SMILES are checked against the campaign's accumulated set; duplicates are discarded.
3. **Repair**: Molecules receiving a *Fix* routing verdict (one correctable structural defect, e.g. a single violated property window) are fed back into the next generation prompt with their failure reasons, allowing the LLM to apply a targeted structural correction rather than generating from scratch. Only one repair attempt is made per molecule; if the corrected structure still fails, it is discarded.
4. **Invalid SMILES rate**: Across all experiments (Contributions 1-4), the post-hoc evaluator reported 100% valid SMILES for all conditions and targets, indicating that the combination of explicit validity

rules in the prompt and RDKit post-processing effectively eliminates chemically invalid output before it reaches the cascade.

13 STRUCTURAL TRANSPARENCY OF PRIORITIZED CANDIDATES

This section makes the raw candidate data directly inspectable at the molecule level. Tables S8-S10, S11, and S12-S13 report, for all 35 advanced candidates from Experiment 1 (Contribution 1; EGFR $n = 22$, KRAS^{G12C} $n = 4$, BRD4 $n = 9$), the 2D structure, canonical SMILES, AutoDock Vina docking score, composite score, ADMET flag (PASS = no warning-level hERG or AMES liability; WARN = hERG ≥ 0.50 or AMES ≥ 0.50), Bemis-Murcko scaffold ID, and the nearest known PubChem compound (via a similarity search executed at decreasing thresholds, 95% down to 70%, until a hit was returned), the Tanimoto similarity of each candidate to that nearest neighbor, and whether the candidate's own Bemis-Murcko scaffold exactly matches the neighbor's scaffold. This allows readers to assess whether generated chemical matter represents close lead-optimization analogues of known compounds versus genuinely novel chemical space. Across all three targets, 24 of the 35 advanced candidates (68.6%) share a Bemis-Murcko scaffold with their nearest known PubChem neighbor once stereochemistry and tautomeric representation are normalized (see methodology below): 15 of 22 EGFR candidates (68.2%), 1 of 4 KRAS^{G12C} candidates (25.0%), and 8 of 9 BRD4 candidates (88.9%). This pattern tracks each target's literature density: the BRD4 thieno-triazolo-diazepine series (candidates 4-9) matches its nearest PubChem neighbor's scaffold in all six cases, several EGFR candidates built on the quinazoline (erlotinib-like) or triazol-3(4H)-one-aminopyrimidine scaffolds show Tanimoto $\geq$0.8 to known PubChem entries with matching scaffolds, and the diarylamide/benzanilide-class KRAS^{G12C} candidates, drawn from this target's comparatively sparse literature coverage, retain only one scaffold match. Two candidates are confirmed full structural duplicates of existing PubChem compounds by full-molecule InChIKey comparison, and six additional candidates match at the connectivity layer (see below).

Nearest-neighbor methodology. The nearest-neighbor analysis was performed post-hoc for transparency and is not part of the platform's in-loop evaluation pipeline. For each candidate, the nearest known PubChem compound was identified via PubChem similarity search, queried at decreasing similarity thresholds (95%, 90%, 80%, 70%) until at least one hit was returned. PubChem's similarity search uses its own 881-bit subgraph fingerprint, which differs from the Morgan/ECFP4 fingerprint used for the Tanimoto values reported in the tables. After retrieving the top candidate CIDs, the Tanimoto similarity between the MolecureAI candidate and each PubChem hit was computed using Morgan fingerprints (radius 2, 2048-bit, ECFP4-equivalent). The hit with the highest Tanimoto was retained as the nearest neighbor.

Two distinct structural comparisons are reported, at two different levels of stringency. **Scaffold match** assesses ring-system identity only: the Bemis-Murcko scaffold of the candidate and of the nearest neighbor were each extracted, stereochemistry was removed from both scaffolds, and the two were compared via InChIKey equality rather than by direct SMILES string comparison. This InChIKey-based comparison is necessary because PubChem's connectivity SMILES omit stereochemical annotations present in the locally generated candidate SMILES, and because scaffold extraction can yield tautomerically distinct but chemically equivalent representations; a raw string comparison of scaffold SMILES is therefore unreliable and was not used for the results reported here. **Full structural identity** is the stricter, separate comparison: the complete, unmodified candidate and nearest-neighbor structures (not just their scaffolds) were compared by full-molecule InChIKey across all three layers (connectivity, stereochemistry, protonation). A scaffold match indicates the candidate shares its core ring system with a known compound; full structural identity indicates the candidate *is* that known compound.

Because the reported Tanimoto is based on a hashed Morgan fingerprint rather than a canonical-structure identifier, a value of 1.00 does not by itself indicate structural identity: two compounds differing in peripheral substituents or stereochemistry can produce identical ECFP4 fingerprints when the differing features hash to the same bit positions or fall outside the fingerprint's resolution. Eight candidates report Tanimoto $= 1.00$ (EGFR 3, 4, 7, 11, 14, 16 and BRD4 4, 7). Under the stereochemistry-normalized scaffold-match procedure described above, all eight show a scaffold match; stereochemistry normalization (stereochemistry and tautomer representation differences between locally generated and PubChem-sourced SMILES) is required for reliable scaffold comparison; without it, scaffold SMILES that are chemically equivalent can appear distinct.

Of these eight, two are confirmed exact structural matches by full-molecule InChIKey comparison against their PubChem nearest neighbor: EGFR 4 (CID 5328234, InChIKey LQIKOEJHMCCOSP-UHFFFAOY SA-N) and EGFR 16 (CID 141885, InChIKey UZFYPOXSNSAIKK-UHFFFAOYSA-N). In these two cases the full three-layer InChIKey (connectivity, stereochemistry, protonation) is identical between the MolecureAI candidate and the PubChem compound, confirming that these are known compounds rather than generated chemical matter. The remaining six, EGFR 3, EGFR 7, EGFR 11, EGFR 14, BRD4 4, and BRD4 7, share the same connectivity layer (first block of the InChIKey) as their PubChem nearest neighbor but differ in the stereochemistry layer. This indicates that the candidate is a specific stereoisomer of a known compound whose PubChem entry has unspecified stereochemistry. All eight are flagged with a dagger marker (†) in the respective tables. Their recovery, concentrated on the most densely represented EGFR and BRD4 chemotypes in the medicinal-chemistry literature, is consistent with the target-space saturation discussed in Section 4.3 of the main text.

ChEMBL cross-validation of Tanimoto $=$ 1.00 candidates. Each of these eight candidates was cross-referenced against the ChEMBL bioactivity database by InChIKey lookup. This post-hoc cross-validation is distinct from the platform's internal novelty gate, which checks candidates against ChEMBL compounds with documented IC50 $\leq$ 100 nM for the target, FDA-approved drugs, and hardcoded reference inhibitors *before* any candidate is retained. The post-hoc PubChem nearest-neighbor analysis reported here, by contrast, searches all $\sim$119 million PubChem compounds regardless of bioactivity and serves a transparency function, not a filtering one.

The ChEMBL cross-validation results are summarized in Table S7. Four categories emerge, forming a clean 2×2 partition on match stringency (Full vs. Connectivity) and bioactivity documentation:

1. *Full structural match with documented target activity* (1 candidate): EGFR 4 (CID 5328234, CHEMBL176582) is a full three-layer InChIKey match to its PubChem nearest neighbor and is a documented EGFR inhibitor with IC50 = 0.01 nM.
2. *Connectivity-layer match with documented activity on the campaign target* (3 candidates): EGFR 7 (CID 136286736, CHEMBL5073622) matches its PubChem nearest neighbor at the connectivity layer and is documented in ChEMBL as a dual EGFR/BTK inhibitor (EGFR IC50$\approx$5 nM, BTK IC50 = 2 nM). BRD4 4 (CID 172471234, CHEMBL5440537) and BRD4 7 (CID 172471837, CHEMBL5440963) likewise match at the connectivity layer and are documented BRD4 inhibitors (IC50 = 0.2-5.0 nM and 0.3-10.0 nM, respectively). All three differ from their PubChem neighbor only in stereochemistry.
3. *Connectivity-layer match without target-specific bioactivity* (3 candidates): EGFR 3 (CID 136295072, CHEMBL5083885) is present in ChEMBL but only as a BTK inhibitor (IC50 = 31.6 nM), not an EGFR inhibitor. EGFR 11 (CID 136286772, CHEMBL6033192) and EGFR 14 (CID 136295068, CHEMBL5987783) are similarly present in ChEMBL only as BTK inhibitors (IC50 = 6.3 and

1.3 nM respectively), not EGFR inhibitors. All three match their PubChem nearest neighbor at the connectivity layer, differing only in stereochemistry. Since the pipeline's blocklist is target-specific (EGFR IC50 $\leq$ 100 nM), these compounds were correctly not blocked.

4. *Full structural match without bioactivity data* (1 candidate): EGFR 16 (CID 141885) is a full three-layer InChIKey match to its PubChem nearest neighbor but is absent from ChEMBL entirely; no bioactivity data are available in either database.

Table S7. ChEMBL cross-validation of the eight Tanimoto = 1.00 candidates. "Full" = identical full-molecule InChIKey (all three layers); "Connectivity" = same connectivity layer (first InChIKey block), stereochemistry layer differs.

Candidate	CID	ChEMBL ID	Target activity	InChIKey	Match
EGFR 3	136295072	CHEMBL5083885	BTK IC50=31.6 nM (not EGFR)	YFGTVWDQSBQMBM-LBPRGKRZSA-N	Connectivity
EGFR 4	5328234	CHEMBL176582	**EGFR IC50=0.01 nM**	LQIKOEJHMCCOSP-UHFFFAOYSA-N	Full
EGFR 7	136286736	CHEMBL5073622	EGFR IC50≈5 nM	HIMUHMBGRATXMK-LBPRGKRZSA-N	Connectivity
EGFR 11	136286772	CHEMBL6033192	BTK IC50=6.3 nM (not EGFR)	MTCIVWJQXPPNAS-LBPRGKRZSA-N	Connectivity
EGFR 14	136295068	CHEMBL5987783	BTK IC50=1.3 nM (not EGFR)	OUXQVZPHPUFGSB-LBPRGKRZSA-N	Connectivity
EGFR 16	141885	Not in ChEMBL	N/A	UZFYPOXSNSAIKK-UHFFFAOYSA-N	Full
BRD4 4	172471234	CHEMBL5440537	**BRD4 IC50=0.2-5.0 nM**	DBKJPAGKXDFFCG-SFHVURJKSA-N	Connectivity
BRD4 7	172471837	CHEMBL5440963	**BRD4 IC50=0.3-10.0 nM**	XCLRZZASCPTRLU-KRWDZBQOSA-N	Connectivity

Fourteen candidates report Tanimoto values below 0.70 (ranging from 0.17 to 0.67); the lowest PubChem search threshold (70%) applies to PubChem's own 881-bit subgraph fingerprint, not to the Morgan fingerprint, so a compound satisfying PubChem's $\geq$70% criterion can yield a substantially lower local Tanimoto. These cases represent the most distant nearest neighbors returned by PubChem and indicate that the candidate lies in a relatively less populated region of chemical space for that target.

Table S8. Structural transparency table for EGFR advanced candidates (n=22, part 1/3): 2D structure, SMILES, docking and composite scores, ADMET flags (PASS = no warning-level liability; WARN = hERG $\geq$ 0.50 or AMES $\geq$ 0.50), Bemis-Murcko scaffold ID, and nearest known PubChem analogue with Tanimoto similarity and scaffold-match status. Continued in Table S9.

Struct.	SMILES	Dock.	Comp.	ADMET	Scaf.	Nearest PubChem	Tanimoto (scaf. match)
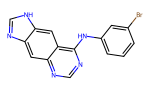	Nc1ncnc2oc(-c3cccc(C(F)F)c3)cc12	-8.07	0.684	N/A	S9	CID 696524	0.66 (yes)
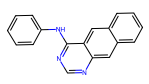	c1ccc(Nc2ncnc3cc4ccccc4cc23)cc1	-9.37	0.657	WARN	S2	CID 606939	0.53 (no)
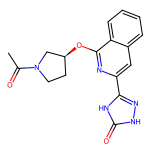	CC(=O)N1CC[C@H](Oc2nc(-c3n[nH]c(=O)[nH]3)cc3ccccc23)C1	-8.76	0.710	PASS	S3	CID 136295072	1.00 (yes)†
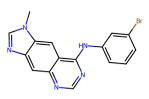	Cn1cnc2cc3ncnc(Nc4cccc(Br)c4)c3cc21	-8.57	0.649	WARN	S1	CID 5328234	1.00 (yes)†
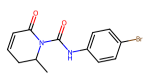	CC1CC=CC(=O)N1C(=O)Nc1ccc(Br)cc1	-7.47	0.686	PASS	S4	CID 735609	0.36 (no)
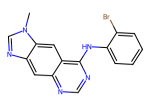	Cn1cnc2cc3ncnc(Nc4ccccc4Br)c3cc21	-8.17	0.624	WARN	S1	CID 5328234	0.67 (yes)
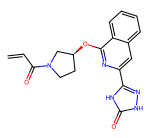	C=CC(=O)N1CC[C@H](Oc2nc(-c3n[nH]c(=O)[nH]3)cc3ccccc23)C1	-8.63	0.693	PASS	S3	CID 136286736	1.00 (yes)†
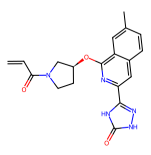	C=CC(=O)N1CC[C@H](Oc2nc(-c3n[nH]c(=O)[nH]3)cc3ccc(C)cc23)C1	-9.18	0.701	PASS	S3	CID 136286772	0.81 (yes)

Table S9. Structural transparency table for EGFR advanced candidates (n=22, part 2/3; continued from Table S8): 2D structure, SMILES, docking and composite scores, ADMET flags (PASS = no warning-level liability; WARN = hERG $\geq$ 0.50 or AMES $\geq$ 0.50), Bemis-Murcko scaffold ID, and nearest known PubChem analogue with Tanimoto similarity and scaffold-match status. Continued in Table S10.

Struct.	SMILES	Dock.	Comp.	ADMET	Scaf.	Nearest PubChem	Tanimoto (scaf. match)
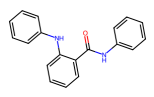	O=C(Nc1ccccc1)c1ccccc1Nc1ccccc1	-8.43	0.689	WARN	S5	CID 2546421	0.81 (yes)
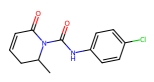	CC1CC=CC(=O)N1C(=O)Nc1ccc(Cl)cc1	-7.84	0.701	PASS	S4	CID 45832543	0.39 (no)
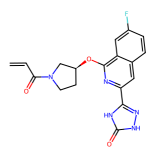	C=CC(=O)N1CC[C@H](Oc2nc(-c3n[nH]c(=O)[nH]3)cc3ccc(F)cc23)C1	-8.97	0.701	PASS	S3	CID 136286772	1.00 (yes)†
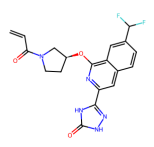	C=CC(=O)N1CC[C@H](Oc2nc(-c3n[nH]c(=O)[nH]3)cc3ccc(C(F)F)cc23)C1	-8.81	0.680	PASS	S3	CID 136286772	0.80 (yes)
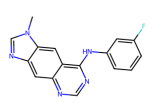	Cn1cnc2cc3ncnc(Nc4cccc(F)c4)c3cc21	-8.39	0.635	WARN	S1	CID 11163155	0.59 (no)
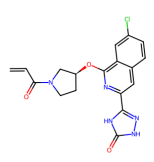	C=CC(=O)N1CC[C@H](Oc2nc(-c3n[nH]c(=O)[nH]3)cc3ccc(Cl)cc23)C1	-8.81	0.688	PASS	S3	CID 136295068	1.00 (yes)†
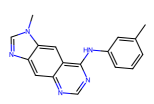	Cc1cccc(Nc2ncnc3cc4ncn(C)c4cc23)c1	-8.59	0.645	WARN	S1	CID 25198680	0.39 (yes)

Table S10. Structural transparency table for EGFR advanced candidates (n=22, part 3/3; continued from Table S9): 2D structure, SMILES, docking and composite scores, ADMET flags (PASS = no warning-level liability; WARN = hERG $\geq$ 0.50 or AMES $\geq$ 0.50), Bemis-Murcko scaffold ID, and nearest known PubChem analogue with Tanimoto similarity and scaffold-match status.

Struct.	SMILES	Dock.	Comp.	ADMET	Scaf.	Nearest PubChem	Tanimoto (scaf. match)
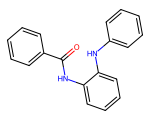	`O=C(Nc1ccccc1Nc1ccccc1)c1ccccc1`	-8.58	0.684	WARN	S6	CID 141885	1.00 (yes)†
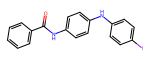	`O=C(Nc1ccc(Nc2ccc(I)cc2)cc1)c1ccccc1`	-9.45	0.660	WARN	S7	CID 681265	0.79 (yes)
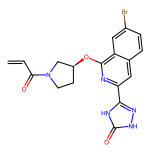	`C=CC(=O)N1CC[C@H](Oc2nc(-c3n[nH]c(=O)[nH]3)cc3ccc(Br)cc23)C1`	-8.40	0.672	PASS	S3	CID 136286772	0.81 (yes)
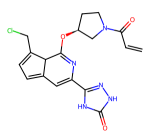	`C=CC(=O)N1CC[C@H](OC2=NC(c3n[nH]c(=O)[nH]3)=CC3=CC=C(CCl)C32)C1`	-8.48	0.666	WARN	S3	CID 166196858	0.17 (no)
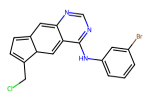	`ClCC1=CC=C2C=c3ncnc(Nc4cccc(Br)c4)c3=CC21`	-9.40	0.711	WARN	S8	CID 6605245	0.32 (no)
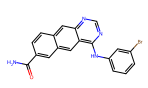	`NC(=O)c1ccc2cc3ncnc(Nc4cccc(Br)c4)c3cc2c1`	-8.84	0.662	WARN	S2	CID 151919267	0.54 (no)
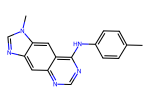	`Cc1ccc(Nc2ncnc3cc4ncn(C)c4cc23)cc1`	-8.16	0.626	WARN	S1	CID 25198680	0.51 (yes)

†Structural match to an existing PubChem compound: full three-layer InChIKey identity (connectivity, stereochemistry, protonation) for EGFR 4 and EGFR 16; connectivity-layer identity (stereochemistry layer differs) for EGFR 3, 7, 11, 14 and BRD4 4, 7. See Section 13 methodology and ChEMBL cross-validation table for details.

EGFR scaffold legend: S1 = 4-anilinoquinazoline (`c1ccc(Nc2ncnc3cc4nc[nH]c4cc23)cc1`); S2 = naphthyridine-aminopyrimidine (`c1ccc(Nc2ncnc3cc4ccccc4cc23)cc1`); S3 = triazol-3(4H)-one-aminopyrimidine (`O=c1[nH]nc(-c2cc3ccccc3c(O[C@H]3CCNC3)n2)[nH]1`); S4 = piperidone-urea (`O=C1C=CCCN1C(=O)Nc1ccccc1`); S5 = benzanilide (`O=C(Nc1ccccc1)c1ccccc1Nc1ccccc1`); S6 = diarylamine-benzanilide (`O=C(Nc1ccccc1Nc1ccccc1)c1ccccc1`);

S7 = diarylamine-benzamide (O=C(Nc1ccc(Nc2ccccc2)cc1)c1ccccc1); S8 = chloromethyl-quinazoline (C1=CC2C=c3c(Nc4ccccc4)ncnc3=CC2=C1); S9 = furo[3,2-d]pyrimidine (c1ccc(-c2cc3cncnc3o2)cc1).

Table S11. Structural transparency table for KRAS^{G12C} advanced candidates (n=4): 2D structure, SMILES, docking and composite scores, ADMET flags (PASS = no warning-level liability; WARN = hERG $\geq$ 0.50 or AMES $\geq$ 0.50), Bemis-Murcko scaffold ID, and nearest known PubChem analogue with Tanimoto similarity and scaffold-match status.

Struct.	SMILES	Dock.	Comp.	ADMET	Scaf.	Nearest PubChem	Tanimoto (scaf. match)
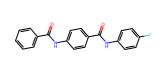	O=C(Nc1ccc(C(=O)Nc2ccc(F)cc2)cc1)c1ccccc1	-8.92	0.722	PASS	S1	CID 166633728	0.78 (no)
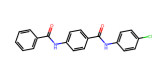	O=C(Nc1ccc(C(=O)Nc2ccc(Cl)cc2)cc1)c1ccccc1	-8.83	0.708	PASS	S1	CID 1217064	0.73 (yes)
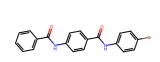	O=C(Nc1ccc(C(=O)Nc2ccc(Br)cc2)cc1)c1ccccc1	-8.80	0.696	PASS	S1	CID 869239	0.71 (no)
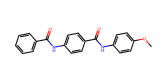	COc1ccc(NC(=O)c2ccc(NC(=O)c3ccccc3)cc2)cc1	-8.49	0.670	PASS	S1	CID 138944	0.86 (no)

KRAS^{G12C} scaffold legend: S1 = diarylamide (O=C(Nc1ccc(C(=O)Nc2ccccc2)cc1)c1ccccc1).

Table S12. Structural transparency table for BRD4 advanced candidates (n=9, part 1/2): 2D structure, SMILES, docking and composite scores, ADMET flags (PASS = no warning-level liability; WARN = hERG $\geq$ 0.50 or AMES $\geq$ 0.50), Bemis-Murcko scaffold ID, and nearest known PubChem analogue with Tanimoto similarity and scaffold-match status. Continued in Table S13.

Struct.	SMILES	Dock.	Comp.	ADMET	Scaf.	Nearest PubChem	Tanimoto (scaf. match)
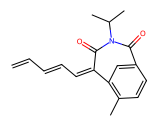	`C=CC=CC=c1c(=O)n(C(C)C)c(=O)c2ccc(C)c1c2`	-7.20	0.558	PASS	S5	CID 897448	0.22 (no)
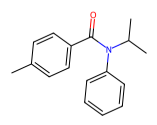	`Cc1ccc(C(=O)N(c2ccccc2)C(C)C)cc1`	-5.33	0.572	PASS	S1	CID 770767	0.62 (yes)
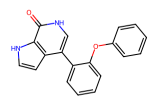	`O=c1[nH]cc(-c2ccccc2Oc2ccccc2)c2cc[nH]c12`	-5.75	0.593	WARN	S2	CID 71616297	0.62 (yes)
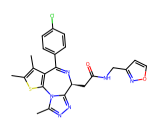	`Cc1sc2c(c1C)C(c1ccc(Cl)cc1)=N[C@@H](CC(=O)NCc1ccon1)c1nnc(C)n1-2`	-6.26	0.578	WARN	S3	CID 172471234	1.00 (yes)†
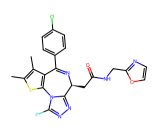	`Cc1sc2c(c1C)C(c1ccc(Cl)cc1)=N[C@@H](CC(=O)NCc1ncco1)c1nnc(F)n1-2`	-6.32	0.581	WARN	S4	CID 172471837	0.89 (yes)

Table S13. Structural transparency table for BRD4 advanced candidates (n=9, part 2/2; continued from Table S12): 2D structure, SMILES, docking and composite scores, ADMET flags (PASS = no warning-level liability; WARN = hERG $\geq$ 0.50 or AMES $\geq$ 0.50), Bemis-Murcko scaffold ID, and nearest known PubChem analogue with Tanimoto similarity and scaffold-match status.

Struct.	SMILES	Dock.	Comp.	ADMET	Scaf.	Nearest PubChem	Tanimoto (scaf. match)
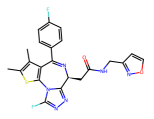	`Cc1sc2c(c1C)C(c1ccc(F)cc1)=N[C@@H](CC(=O)NCc1ccon1)c1nnc(F)n1-2`	-6.28	0.580	WARN	S3	CID 172471234	0.75 (yes)
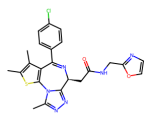	`Cc1sc2c(c1C)C(c1ccc(Cl)cc1)=N[C@@H](CC(=O)NCc1ncco1)c1nnc(C)n1-2`	-6.19	0.564	WARN	S4	CID 172471837	1.00 (yes)†
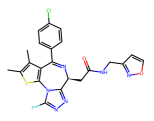	`Cc1sc2c(c1C)C(c1ccc(Cl)cc1)=N[C@@H](CC(=O)NCc1ccon1)c1nnc(F)n1-2`	-6.33	0.584	WARN	S3	CID 172471234	0.85 (yes)
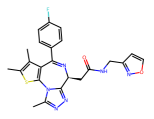	`Cc1sc2c(c1C)C(c1ccc(F)cc1)=N[C@@H](CC(=O)NCc1ccon1)c1nnc(C)n1-2`	-6.41	0.591	WARN	S3	CID 172471234	0.85 (yes)

BRD4 scaffold legend: S1 = benzanilide (`O=C(Nc1ccccc1)c1ccccc1`); S2 = diarylfuran-pyrroloquinoline (`O=c1[nH]cc(-c2ccccc2Oc2ccccc2)c2cc[nH]c12`); S3 = thieno-triazolo-diazepine (isoxazole variant) (`O=C(C[C@@H]1N=C(c2ccccc2)c2ccsc2-n2cnnc21)NCc1ccon1`); S4 = thieno-triazolo-diazepine (oxazole variant) (`O=C(C[C@@H]1N=C(c2ccccc2)c2ccsc2-n2cnnc21)NCc1ncco1`); S5 = phthalimide-alkenyl (`C=CC=CC=c1c(=O)n(C(C)C)c(=O)c2ccc(C)c1c2`).
